# Evaluation of chronic drug-induced electrophysiological and cytotoxic effects using human-induced pluripotent stem cell-derived cardiomyocytes (hiPSC-CMs)

**DOI:** 10.3389/fphar.2023.1229960

**Published:** 2023-07-10

**Authors:** C. Altrocchi, K. Van Ammel, M. Steemans, M. Kreir, F. Tekle, A. Teisman, D. J. Gallacher, H. R. Lu

**Affiliations:** ^1^ A Division of Janssen Pharmaceutica NV, Global Safety Pharmacology, Preclinical Sciences and Translational Safety, Janssen R&D, Beerse, Belgium; ^2^ A Division of Janssen Pharmaceutica NV, Cell Health Assessment Group, Preclinical Sciences and Translational Safety, Janssen R&D, Beerse, Belgium; ^3^ A Division of Janssen Pharmaceutica NV, Statistics and Decision Sciences, Global Development, Janssen R&D, Beerse, Belgium

**Keywords:** drug-induced cardiotoxicity, hiPSC-CMs, *in vitro* assay, arrhythmias, cytotoxicity, risk prediction, multielectrode arrays (MEA)

## Abstract

**Introduction:** Cardiotoxicity is one of the leading causes of compound attrition during drug development. Most *in vitro* screening platforms aim at detecting acute cardio-electrophysiological changes and drug-induced chronic functional alterations are often not studied in the early stage of drug development. Therefore, we developed an assay using human-induced pluripotent stem cell-derived cardiomyocytes (hiPSC-CMs) that evaluates both drug-induced acute and delayed electrophysiological and cytotoxic effects of reference compounds with clinically known cardiac outcomes.

**Methods:** hiPSC-CMs were seeded in 48-well multielectrode array (MEA) plates and were treated with four doses of reference compounds (covering and exceeding clinical free plasma peak concentrations -fC_max_ values) and MEA recordings were conducted for 4 days. Functional-electrophysiological (field-potentials) and viability (impedance) parameters were recorded with a MEA machine.

**Results:** To assess this platform, we tested tyrosine-kinase inhibitors with high-cardiac risk profile (sunitinib, vandetanib and nilotinib) and low-cardiac risk (erlotinib), as well as known classic cardiac toxic drugs (doxorubicin and BMS-986094), ion-channel trafficking inhibitors (pentamidine, probucol and arsenic trioxide) and compounds without known clinical cardiotoxicity (amoxicillin, cetirizine, captopril and aspirin). By evaluating the effects of these compounds on MEA parameters, the assay was mostly able to recapitulate different drug-induced cardiotoxicities, represented by a prolongation of the field potential, changes in beating rate and presence of arrhythmic events in acute (<2 h) or delayed phase ≥24 h, and/or reduction of impedance during the delayed phase (≥24 h). Furthermore, a few reference compounds were tested in hiPSC-CMs using fluorescence- and luminescence-based plate reader assays, confirming the presence or absence of cytotoxic effects, linked to changes of the impedance parameters measured in the MEA assay. Of note, some cardiotoxic effects could not be identified at acute time points (<2 h) but were clearly detected after 24 h, reinforcing the importance of chronic drug evaluation.

**Discussion:** In conclusion, the evaluation of chronic drug-induced cardiotoxicity using a hiPSC-CMs *in vitro* assay can contribute to the early de-risking of compounds and help optimize the drug development process.

## 1 Introduction

Cardiotoxicity is one of the leading causes of drug withdrawal from the market (Laverty et al., 2011; Weaver and Valentin, 2018; Mamoshina et al., 2021). Concerns about cardiovascular safety have led to a substantial reduction in drug approvals from different therapeutic areas in the last decade, leading to a sharp increase in costs associated with such failure (Ferdinandy et al., 2019). Mechanisms behind drug-induced cardiotoxicity are multiple and not yet completely understood. One of the most common and well-investigated aspects of cardiotoxicity is drug-induced prolongation of the QT_c_ interval of the electrocardiogram (ECG). Drug-induced long QT syndrome is caused by an alteration of the action potential in cardiomyocytes, most often linked to repolarization prolongation due to inhibition of the human Ether-à-go-go-Related Gene (hERG) encoded delayed rectifier potassium channel (I_Kr_), which can lead to life-threatening cardiac arrhythmias, such as Torsade de Pointes (TdP) (Tisdale et al., 2020). Furthermore, impairment of structural integrity and cardiac dysfunction also represent common cardiotoxic effects, such as in the seminal case of the hepatitis C drug BMS-986094, which induced heart failure in patients (Ahmad et al., 2015). In some circumstances, drug-induced cardiotoxicity might only occur upon chronic drug treatments, such as in the case of anthracycline treatment for cancer. Doxorubicin, one of the most commonly used anthracyclines in the treatment of hematologic and breast malignancies, can induce heart failure months to years after administration (Wenningmann et al., 2019). Compounds can also affect the trafficking, assembly and function of key proteins including ion channels (e.g., hERG channel) in cardiomyocytes. A representative drug that impairs hERG trafficking is pentamidine, an antiprotozoal agent used to treat trypanosomiasis, leishmaniasis and *Pneumocystis carinii* pneumonia in HIV-infected patients (Rybniker et al., 2010). Cases of QT_c_ prolongation and even TdP were reported to occur only after several days following intravenous administration of pentamidine (Eisenhauer et al., 1994; Girgis et al., 1997). Subsequent mechanistic studies determined that pentamidine inhibits the trafficking and maturation of hERG, with an IC_50_ close to therapeutic doses, upon 24 h incubation in hERG-expressing HEK293 cells (Kuryshev et al., 2005). Arsenic trioxide (As_2_O_3_) is a naturally occurring metalloid used in the treatment of relapsed or refractory acute promyelocytic leukemia (Zhu et al., 2019). Environmental or clinical exposure to arsenic can lead to serious side effects, such as ventricular arrhythmias (including TdP), and sudden cardiac death (Barbey and Soignet, 2001; Westervelt et al., 2001). As shown in guinea-pig ventricular myocytes, As_2_O_3_ reduces the membrane expression of hERG and, therefore, prolongs the action-potential duration at clinically relevant concentrations, thus causing cardiotoxicity (Ficker et al., 2004).

In view of the effort of reducing the number of animal studies (in line with the 3Rs -Replacement, Reduction and Refinement-principles) (Han, 2023), the use of *in vitro* models to predict cardiotoxicity has grown exponentially (Narkar et al., 2022). Human-induced pluripotent stem cell-derived cardiomyocytes (hiPSC-CMs) were able to successfully recapitulate the acute and chronic drug-induced cardiotoxicity of many classes of compounds. For example, extensive characterization of doxorubicin-induced cardiotoxicity was conducted and found to be recapitulated in hiPSC-CMs, showing good translational value of *in vitro* studies (Wenningmann et al., 2019). Another class of anticancer drugs linked to cardiotoxicity are tyrosine-kinase inhibitors (TKi). TKi are small molecules that inhibit the phosphorylation activity of hyperactive receptor tyrosine kinases, often overexpressed in cancer cells (Brandão et al., 2022). TKi-induced cardiotoxicity does not appear to be a class-specific effect, with some compounds linked to adverse outcomes in patients (e.g., sunitinib, nilotinib), whilst others have a relatively safer profile (e.g., erlotinib). While the mechanisms behind this are not yet completely understood, hiPSC-CMs recapitulated the phenotype expected from toxicities in man, as shown by Sharma et al. with a panel of 21 FDA-approved TKi (Sharma et al., 2018).

Thanks to the relatively good versatility of hiPSC-CMs, diverse technologies have been applied to investigate drug-induced cardiotoxicity *in vitro* (Guo et al., 2015). For example, functional recordings of hiPSC-CMs electrophysiological parameters and Ca^2+^-handling can be conducted through voltage- and Ca^2+^-sensitive dye measurements. Fluorescent dye-based recordings work particularly well for acute measurement but show some limitations in a chronic setting and with repeated measure studies (Kopljar et al., 2018). Motion field imaging (Kopljar et al., 2017) and impedance-based assays (Guo et al., 2018) have been demonstrated to detect the delayed effect of compounds on hiPSC-CMs, but these platforms provide an index of contractile function and no information on the underlying electrophysiological effects of test compounds. multielectrode array (MEA) platforms allow the recordings of the electrical waveform signals (field potential, FP) generated and shaped by monolayers of hiPSC-CMs (Sala et al., 2017). The FP contour correlates with the cardiac AP and, to some extent, with body surface electrocardiogram (ECG) recordings (Tertoolen et al., 2018). One of the main advantages of the MEA is the possibility of performing repeated measurements in a non-invasive way in a medium/high throughput fashion, rendering it ideal to investigate electrophysiological alterations induced by chronic exposure to compounds. Cytotoxic compounds such as doxorubicin, often cause the complete cessation of beating of hiPSC-CMs monolayers or the disappearance of the repolarization wave (also called T-wave-like repolarization wave analogous to human ECG), impairing the analysis of FP parameters. In the present study, we therefore developed and validated a functional assay that combines electrophysiological recordings of hiPSC-CMs (CDI iCell^2^) by MEA with cytotoxic readouts (impedance), by assessing acute (2 h) and chronic effects (up to 96 h) of thirteen reference compounds. Additionally, we used cytotoxicity assays on hiPSC-CMs, to determine the level of activation of necrotic or apoptotic cell death and to confirm the functional findings on the MEA assay with three reference compounds, with well-known cardiac outcomes in human.

## 2 Materials and methods

### 2.1 Cell culture

A commercially available line of human iPSC-derived cardiomyocytes, iCell-Cardiomyocytes^2^ (Fujifilm Cellular Dynamics Inc., Madison, WI, United States. Donor: 01434, Female, age <18, Lot numbers: 105464, 105170, 105567, and 105998), was used for the current study. The cell donor was registered for ethics committee for research uses (NICHD-NIH, United States with Approval number N-01-HD-4-2865). Cells were seeded according to the instructions of the cell provider, in fibronectin-coated 48-well MEA plates (Axion Cytoview Plates, M768-tMEA-48B) at a density suited to form a monolayer (i.e., 50 K/well) and maintained in culture in a stage incubator (37°C, 5% CO_2_). The baseline recordings were performed 7 days after plating, when hiPSC-CMs formed a well-synchronized beating syncytium and continued for 4 days (96 h). At 48 h, after FP recordings, a 50% medium/compound refreshment was made. Measurements of FPs were performed in CDI Maintenance medium (M1003), containing 10% FBS and supplemented with 30 µM ciprofloxacin [which is not associated, unlike other fluoroquinolones, to QT prolongation (Heemskerk et al., 2017)].

### 2.2 Compounds selection and preparation

At least 4 h before the beginning of the baseline measurements, a full medium exchange was performed (to a final volume of 400 µL per well). Neat compounds were dissolved in 100% DMSO at a stock concentration 1000-fold the highest target concentration. The compound “mother-plate” containing all test concentrations of the compounds (N ≥ 5/concentration) and positive and vehicle controls (N ≥ 4 per plate) at 1000-fold the final concentrations was made on the first day of the assay. Afterwards, these stock solutions were diluted with Maintenance medium (CDi), to 10-fold the intended concentration. The final DMSO concentration in test solutions and vehicle control was 0.1%.

The reference compounds tested in this study were chosen based on reported presence or absence of cardiotoxic effects in humans. The testing concentrations were selected to meet and exceed the free (therapeutic) C_max_ (fC_max_) values reported in literature. The determination of the free therapeutic C_max_ was based on data from FDA-approved drug packages obtained from Elseviers’ PharmaPendium regulatory submission search engine software (www.pharmapendium.com), except for BMS-986094 (as the compound was stopped during development). Table 1 contains further information on the tested compounds, their test concentrations and fC_max_.

**TABLE 1:** List of compounds tested in the study, nominal concentrations, with relative information of free Cmax, CAS (Chemical Abstracts Service) number, catalog, and lot numbers (Cat. And Lot).

Compound name	Doses (µM)	Mechanism of action	Free Cmax (µM)	CAS number	Cat number-Lot number
Aspirin	100, 30, 10, 3	anti-inflammatory and antipyretic drug	11.1	50-78-2	1044006-R059R0
Cetirizine	3, 1, 0.3, 0.1	Histamine-1 antagonist	0.1	83881-52-1	PHR1656-LRAC6703
Amoxicillin	30, 10, 3, 1	penicillin derivative antibiotic	16.1	26787-78-0	A8523-0000122,481
Captopril	30, 10, 3, 1	ACE inhibitor	2.3	62571-86-2	C4042-BCBP9930V
Doxorubicin	3, 1, 0.3, 0.1	Anthracycline-chemotherapic agent	7.4	25316-40-9	A130952-008-2
BMS-986094	10, 3, 1, 0.3	Hepatitis-C antivial drug	N/A	1234490-83-5	19801-5605
Pentamidine	3, 1, 0.3, 0.1	Antiprotozoal medication	0.2	140-64-7	P0547-117K3731
Arsenic Trioxide	3, 1, 0.3, 0.1	chemotherapic agent	0.3	1327-53-3	A1010-BCCD8344
Probucol	10, 3, 1, 0.3	LDL and HDL cholesterol lowering	0.4	23288-49-5	P9672-SLBR2496V
Vandetanib	3, 1, 0.3, 0.1	Chemotherapic agent (tyrosine kinase inhibitor)	0.4	443913-73-3	464332500-A0408668
Sunitinib	1, 0.3, 0.1, 0.03	Chemotherapic agent (tyrosine kinase inhibitor)	0.03	341031-54-7	PZ0012-0000058519
Erlotinib	10, 3, 1, 0.3	Chemotherapic agent (tyrosine kinase inhibitor)	3.9	183321-74-6	CDS022564-1655,157
Nilotinib	1, 0.3, 0.1, 0.01	Chemotherapic agent (tyrosine kinase inhibitor)	0.1	641571-10-0	QA9642-B36606

### 2.3 Data acquisition and analysis

Spontaneous electrical activity was recorded in hiPSC-CMs monolayers as field potentials using the Maestro Pro MEA instrument (Axion Biosystems, Atlanta, GA, United States), which allows the maintenance of accurate temperature and CO_2_ levels (37°C, 5% CO_2_). After the plate was placed in the Maestro Pro and allowed to equilibrate for about 10 min, baseline acquisition of field potential and impedance signals was initiated and lasted for 5 min each. Different time points were chosen for data acquisition: baseline and 2, 24, 48, 72, and 96 h after compounds addition. For the assessment of the effect of compounds on hiPSC-CMs electrophysiology, several parameters were reported: beating rate (BR, measured in beats/minute), repolarization duration (or field-potential duration FPD, measured in ms from the top of the spike to the peak of the “T-wave like” repolarization wave form, (referred to, for simplicity, as “T-wave” in this manuscript) both uncorrected and corrected for beating rate [with Fredericia correction formula, FPDc (Blinova et al., 2018)]. As a readout of cytotoxicity, a background impedance signal was acquired and compared throughout time (Imp, measured in Ohm). As impedance measures resistance of an applied alternating current through the hiPSC-CMs monolayer, a reduction in the attachment of hiPSC-CMs to the bottom of the well due to cell death or changes in the monolayer morphology, causes a drop in the impedance signal.

**FIGURE 1:**
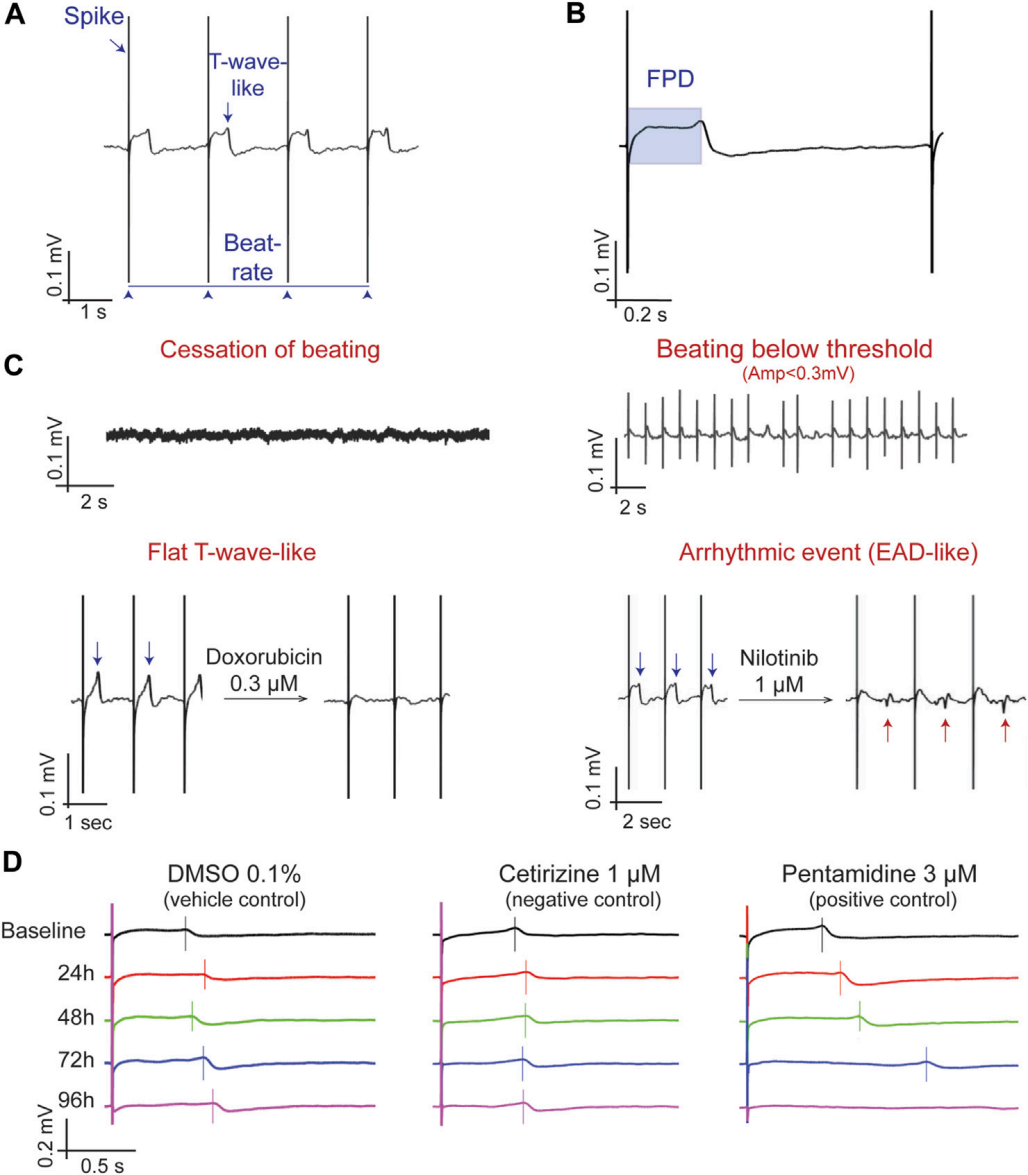
Determination and characterization of the main MEA parameters. **(A)** A typical field potential (FP) recording highlighting the depolarization spike, the T-wave and beating rate. **(B)** Determination of the FP duration (FPD), from the spike to the peak of the T-wave. **(C)** Descriptive parameters for FP changes, including quiescence (i.e., cessation of beating), beating below quantification level (when the monolayer is beating but the field potential amplitude is lower than 0.3 mV), Flat T-wave (absence of a clear repolarization wave), and arrhythmic events (EADs-like events). **(D)** Representative MEA recordings showing the time-dependent changes in the morphology of the FP in DMSO 0.1% (vehicle control), cetirizine 1 µM (negative control) and Pentamidine 3 µM (positive control). The vertical bar is located at the top of the T-wave to mark the FPD; note that at 96 h, pentamidine does not show a repolarization wave (Flat T-wave).

The potential presence of various arrhythmic events was evaluated and noted during the data analysis. Such potential observations include (Figure 1):

• “Early afterdepolarization-like” (EAD-*like*) events [defined as “changes in the morphology or polarity of the repolarization wave,” similar to the description in the CiPA study by Blinova et al. (2018)].
• “Cessation of beating” of the cells (no electrical activity observed).
• “BBQL”: Beats below quantification level (defined as FP amplitude lower than 300 µV).
• “Flat T-wave”: the repolarization wave is not detectable, and thus no measurement of FPD was possible.

After the acquisition of all timepoint measurements, raw data were loaded into the data analysis software Axion Cardiac Analysis Tool (V.3.2.3). As quality control, wells were used for further analysis if they met the following criteria at baseline [already described in (Blinova et al., 2018)]: stable regular beating (coefficient of variation of beat period <5%), FP amplitude ≥300 μV, beat rate ≥18 and ≤90 beats per minute, clear repolarization wave at baseline in >50% of the electrodes and, more than 50% active (covered) electrodes. A.csv file containing information on the FP parameters per well, per time point, was then generated and used as input to the in-house data management system (SPEcII v.3.4, Unlimit-IT, Geel, Belgium), where descriptive statistics were calculated. Summary values at different time points after compound addition are expressed as percent change from baseline and shown as mean ± standard deviation (SD).

### 2.4 Cytotoxicity assays

iCell-Cardiomyocytes^2^ (Fujifilm Cellular Dynamics Inc., Madison, WI, United States) were seeded into 96 well-plates (Greiner Bio One, ref. 655946) according to the manufacturer instructions, at a density of 50 k cells/well. Seven days after seeding, the hiPSC-CMs were used to assess cytotoxic effects of doxorubicin, captopril and sunitinib. To assess effects on viability and cytotoxicity, two assay combination kits were used: 1) RealTime-Glo™ Annexin V Apoptosis and Necrosis Assay [Promega, JA1011 (Kupcho et al., 2019)] and 2) ApoLive-Glo Multiplex Assay [Promega, G6410 (Flörkemeier et al., 2022)]. The assays were performed according to manufacturer instructions. The experiments were repeated at 24, 48, and 72 h in order to assess any time-dependent change in live cells or activation of cell-death pathways.

### 2.5 Statistical analysis

The nonparametric Wilcoxon-Mann Whitney Test was used to compare the percent change from the baseline (Δ%) value between the group treated with the compound at a given concentration and the group treated with the DMSO at each matching time points. A two-sided test at 5% significance level was considered. Hence, the two groups are statistically significant when the *p*-value was less than 0.05. To assess the physiological relevance of changes, a tolerance interval of DMSO experiments was calculated at each time point, for each parameter. Specifically, the Δ% values of all (pooled) DMSO wells (*N* = 81) were used to obtain the non-parametric 80%–95% (*p* = 0.80 & 1-α = 0.95) tolerance interval (TI). TIs indicate an interval where, with a certain confidence level (1- α = 0.95), a specified proportion (*p* = 0.80) of percent change for DMSO data falls. The lower and upper limits based on the TI values were used as cut-offs for the interpretation of the changes. For the cytotoxicity assays, measurements of fluorescence (after background fluorescence subtraction) from wells treated with the compounds were normalized by the average value of vehicle experiments (DMSO 0.1%) to obtain a DMSO-corrected, delta/delta percent change. Experiments from each condition, as delta/delta percent, were averaged and expressed as mean ± SD. Statistical analysis was performed using a non-parametric test (Kruskal-Wallis) and each treatment condition was compared to the relative DMSO 0.1%. Differences were considered statistically significant when the *p*-value was smaller than 0.05.

## 3 Results

### 3.1 Vehicle and negative controls effects on field potential (FP)

**FIGURE 2:**
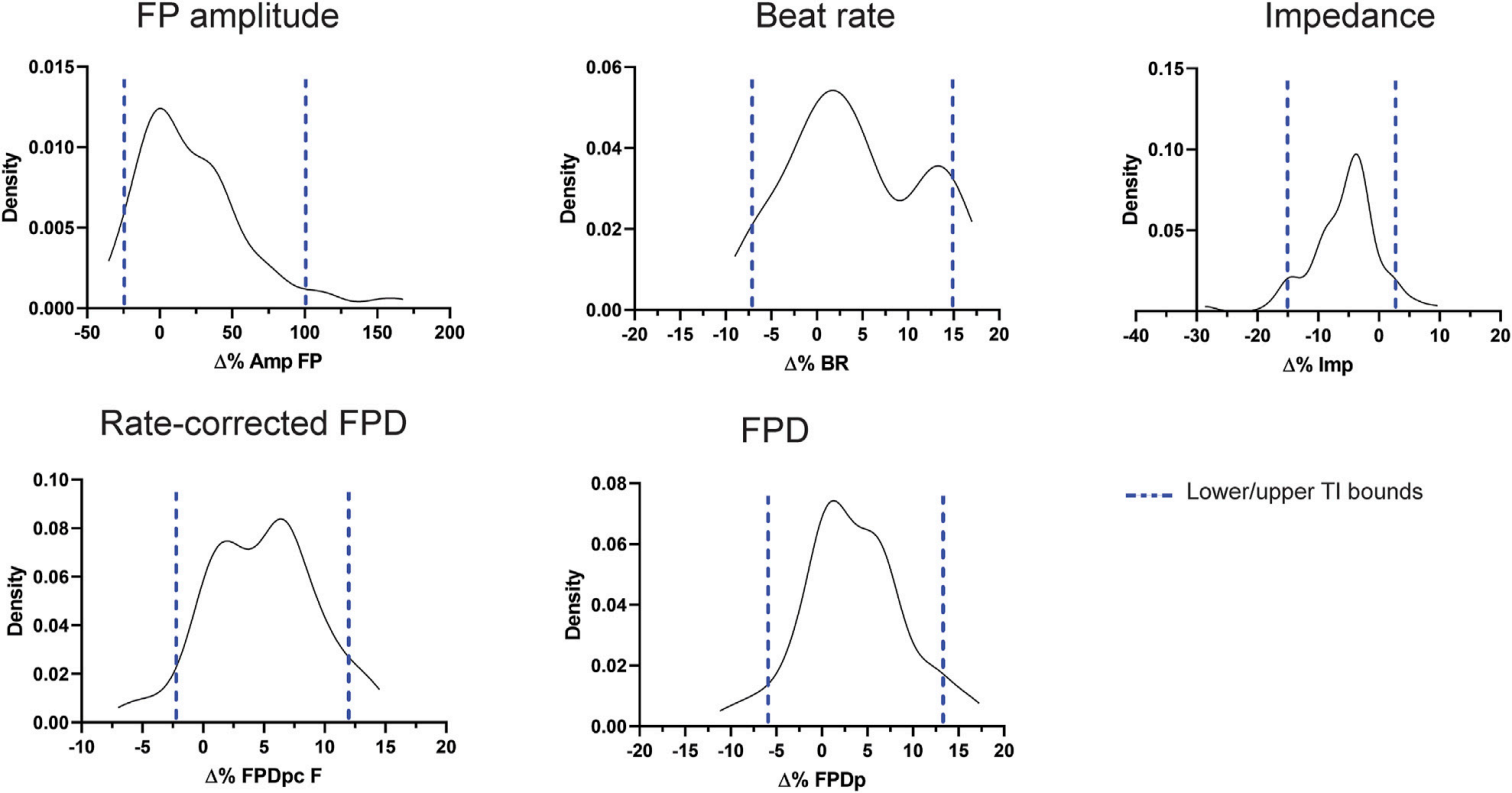
Distribution and tolerance intervals for the main MEA parameters. Density plots depicting the distribution of DMSO 0.1% experiments (expressed as % change from baseline) and the relative tolerance intervals (blue dotted lines) at 24 h.

To understand the variability of the most relevant MEA parameters, we gathered vehicle-treated experiments (DMSO 0.1%) from 15 plates, in which iCell^2^ hiPSC-CMs from four different batches were seeded. Using these data (*n* = 81), we built non-parametric tolerance intervals for five main MEA parameters (Table 2): FP amplitude (Amp FP), beating rate (BR), field-potential duration (FPD), as well as FPD adjusted for beating rate (FPDc) and impedance (Imp), at each of the timepoint (i.e., 2, 24, 48, 72, and 96 h). Figure 2 depicts the distribution of the populations as delta percent over baseline at the 24 h timepoint, for each parameter. The blue dotted lines define the upper and lower bounds of the tolerance intervals. While BR, FPD, FPDc and impedance show a relatively small TI range, the variability of the Amp FP is very large, as the tolerance interval spans from −23.5% to 99.6%, at 24 h. Similar magnitudes were calculated for the remaining time points (Table 2), rendering Amp FP not a useful parameter to assess chronic drug-induce effects. We therefore focused our interpretation of the compounds effect on the other four parameters.

**TABLE 2:** Tolerance interval of DMSO experiments calculated at each time point, for each parameter. The Δ% values of all (pooled) DMSO wells (*N* = 81) were used to obtain the non-parametric 80%–95% (*p* = 0.80 & 1-α = 0.95) tolerance interval (TI). The table includes values of lower and upper bounds, number of replicates used, mean and median for each FP parameter, at all time points.

Parameter	Timepoint h)	Lower bound	Upper bound	Number	Mean	Median
Amp FP	2	−20.4	57.3	81	10.0	5.5
	24	−23.5	99.6	81	23.2	13.1
	48	−36.2	128.8	81	24.8	17.4
	72	−31	133.6	81	36.8	31.6
	96	−38.7	164.9	81	37.5	24.4
BR	2	−7	11	81	1.9	0
	24	−7	16	81	4.4	3
	48	−13	17	81	0.7	0
	72	−13	14	81	−0.7	−2
	96	−24	9	81	−5.8	−7
FPD	2	−5.3	5.8	81	2	2.3
	24	−6.3	13	81	3.4	2.9
	48	−2.4	17.7	81	6.1	6.2
	72	−1.9	20.5	81	6.5	3.9
	96	1.6	29.4	81	10.5	8.6
FPDc	2	−3.3	7.6	81	2.6	2.9
	24	−2.4	12.3	81	4.8	5.3
	48	−0.7	13.2	81	6.1	5.8
	72	−2	16.5	81	5.9	4.9
	96	0.4	20.8	81	7.9	6.9
Imp	2	−7.7	7.7	76	1.3	2.8
	24	−15.2	2.6	82	−5.5	−4.65
	48	−14.2	2.3	82	−6.1	−6.05
	72	−16.1	−1.6	82	−8.9	−8.7
	96	−21.1	1	82	−9.3	−8.85

Amp FP, field potential amplitude; BR, beat rate; FPD: field-potential duration; FPDc: field-potential duration corrected by beat-rate; Imp: impedance.

**FIGURE 3:**
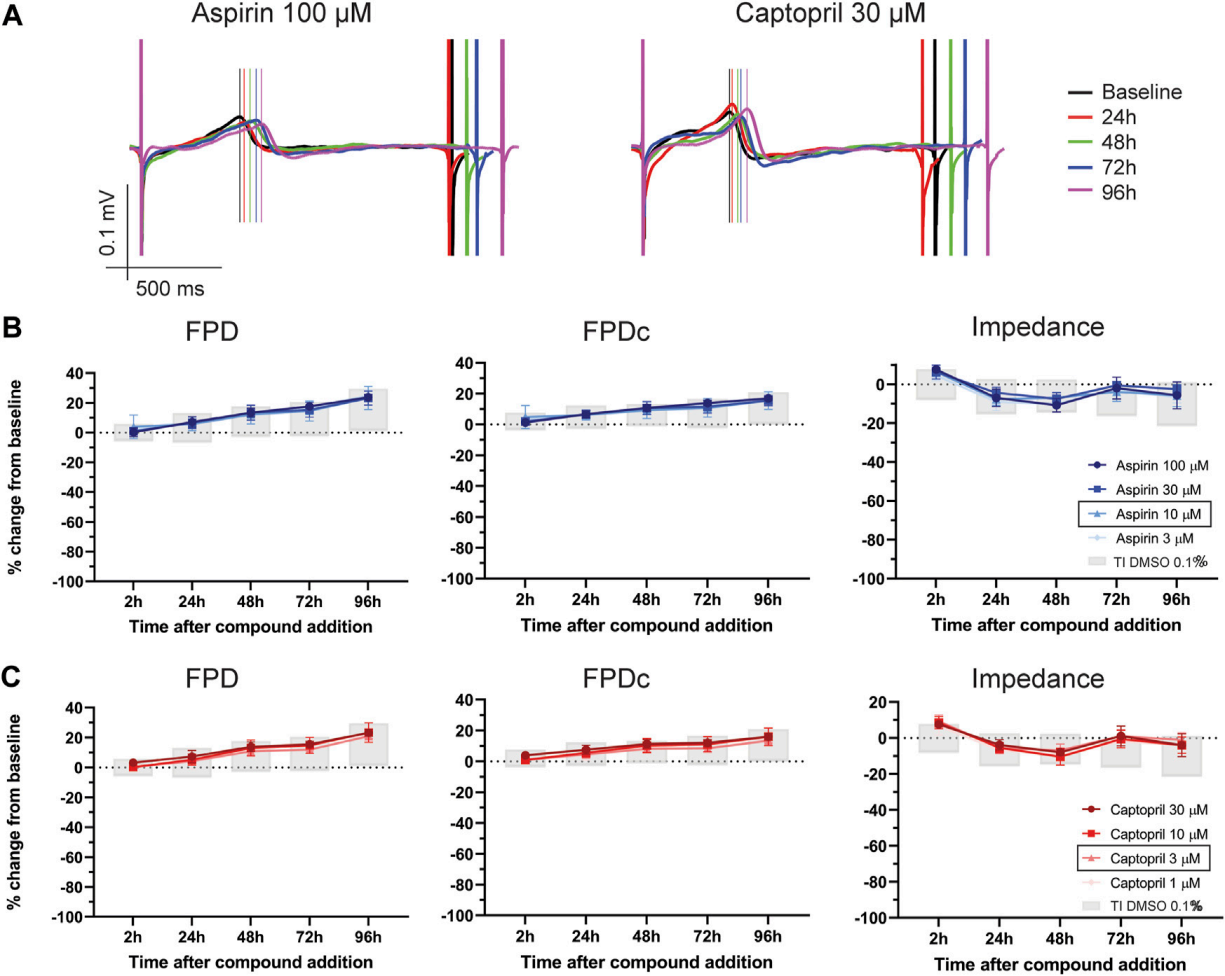
Effect of two negative controls on FP parameters. **(A)** Representative traces of FP from hiPSC-CMs treated with aspirin 100 µM and captopril 30 µM over time (from 24 to 96 h). Notably, no effects on FP morphology were observed. The vertical lines mark the top of the T-wave. Electrophysiological changes in FPD, FPDc and impedance caused by **(B)** aspirin and **(C)** captopril. In the figure legend, the doses indicated with a black outline are the closest to fC_max_.

After the determination of the tolerance intervals for the vehicle control, we tested four compounds from different drug classes, not known to be associated with cardiotoxicities, i.e., amoxicillin, captopril, cetirizine and aspirin. As representative examples, Figure 3A depicts traces of FP in cells treated with aspirin 100 µM and captopril 30 µM over time (24-96 h after compound treatment). Figures 3B, C show the effect of aspirin and captopril, respectively, on FPD, FPDc and impedance. The grey boxes represent the tolerance interval of DMSO 0.1%. As depicted in the figure and described in Supplementary Table S1, the values of all parameters are within the tolerance interval of vehicle experiments, even at the highest concentrations tested, which were 2–30 times the therapeutic fC_max_ values reported in humans (Table 1). Furthermore, no cessation of beating, flat T-wave or arrhythmic-*like* events were detected during the experiments. Aspirin and captopril did not have a significant effect on impedance. The other two negative reference compounds, cetirizine and amoxicillin, also showed similar profile (Supplementary Table S2). However, amoxicillin induced a significant reduction in the beating rate at the latest time point (96 h) at all concentrations, but the values are within the tolerance interval of DMSO 0.1%, therefore not physiologically relevant (Supplementary Figure S1B).

### 3.2 Effect of doxorubicin and BMS-986094 on hiPSC-CMs

**FIGURE 4:**
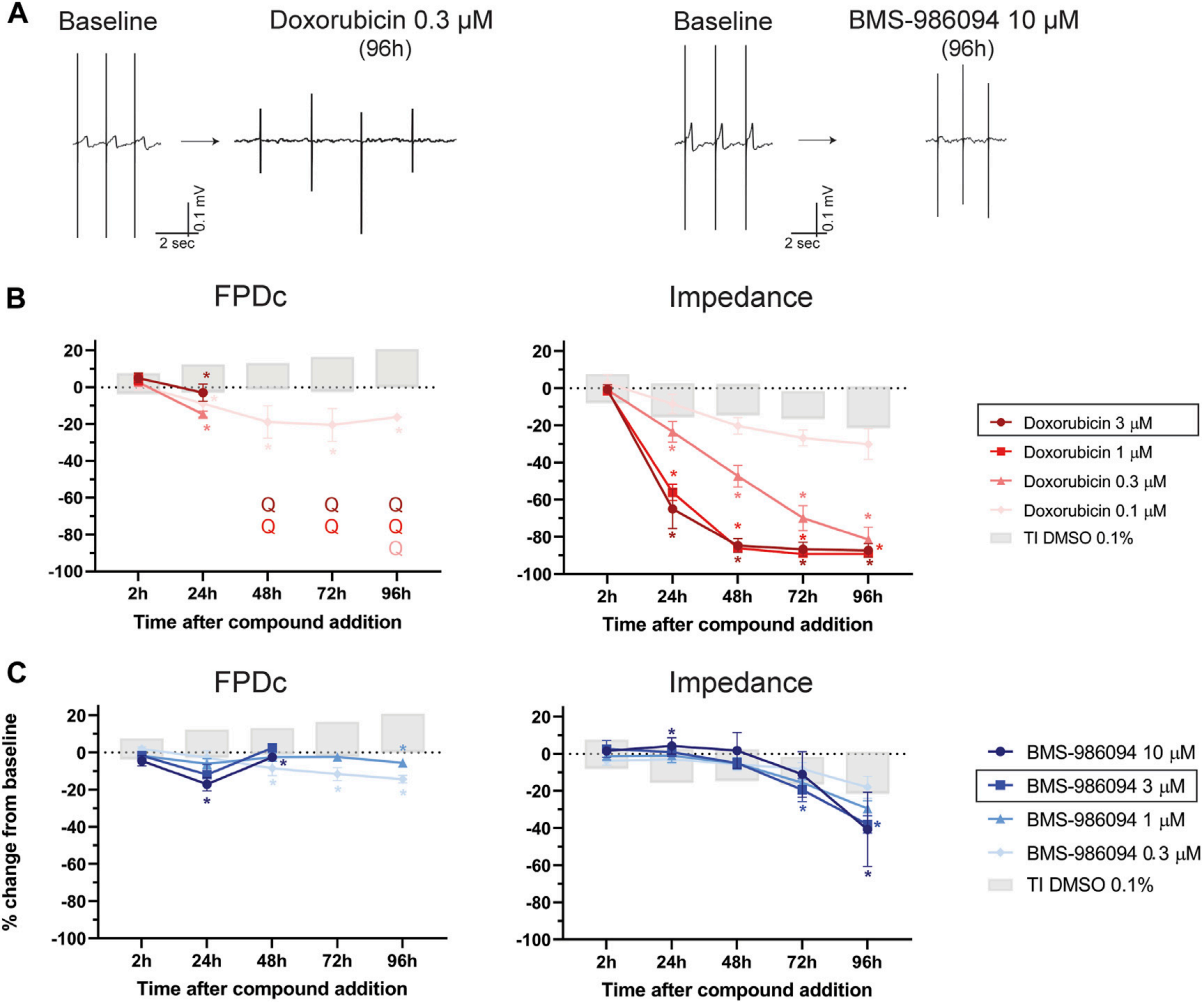
Effect of doxorubicin and BMS-986094 on FP. **(A)** Representative FP trace from baseline and after 96 h-treatment with doxorubicin 0.3 µM or BMS-986094 10 μM, in which the disappearance of the T-wave is evident. Effect of **(B)** doxorubicin and **(C)** BMS-986094 on FPDc and impedance. In the figure legend, the dose of doxorubicin indicated with a black outline is the closest to fC_max_, while that for BMS-986094 is the estimated dose that produced cardiotoxicity in monkeys (Gill et al., 2017). *: *p* < 0.05 and outside the tolerance intervals of vehicle controls.

One of the mechanisms of drug-induced cardiotoxicity is the loss of cardiomyocytes in the heart, which leads to an impairment of cardiac function and heart failure. Doxorubicin is a chemotherapy agent, known to induce long-term cardiac dysfunction in patients and widely used as a tool compound for *in vivo* and *in vitro* studies to model cardiotoxicity (Wenningmann et al., 2019). We therefore evaluated the impact of increasing doses of doxorubicin on hiPSC-CMs. As shown by the representative FP recordings of Figure 4A, and in Figure 4B, doxorubicin caused the disappearance of the T-wave and cessation of beating in all wells, starting at 24 h, at 3 µM and starting at 48 h for the 1 μM and 0.3 µM concentrations (Supplementary Table S7, Supplementary Figure S2). At the lowest concentration (i.e., 0.1 µM) FPDc showed a significant shortening starting at 24 h (Supplementary Table S3). Furthermore, doxorubicin induced a dose-dependent decrease in impedance, which was physiologically relevant for all concentrations (except the lowest 0.1 µM) starting at 24 h, consistent with the already described cytotoxic effects of this drug (Supplementary Table S3).

BMS-986094 is a compound that was intended to be developed to treat hepatitis C, that caused heart failure in patients resulting in termination of its further development. In this assay, BMS-986094 induced an increase in BR (Supplementary Figure S2), shortening of the FPDc at 1 and 0.3 µM and the flattening of the T-wave at 72 h and 96 h at the two highest concentrations (10 and 1 µM), as depicted in Figures 4A, C (Supplementary Tables S3, S7). Furthermore, BMS-986094 had a significant impact on impedance, especially at the last time point (96 h), where it induced a dose-dependent decrease in this parameter (Supplementary Table S3).

### 3.3 Ion channels trafficking inhibitors

**FIGURE 5:**
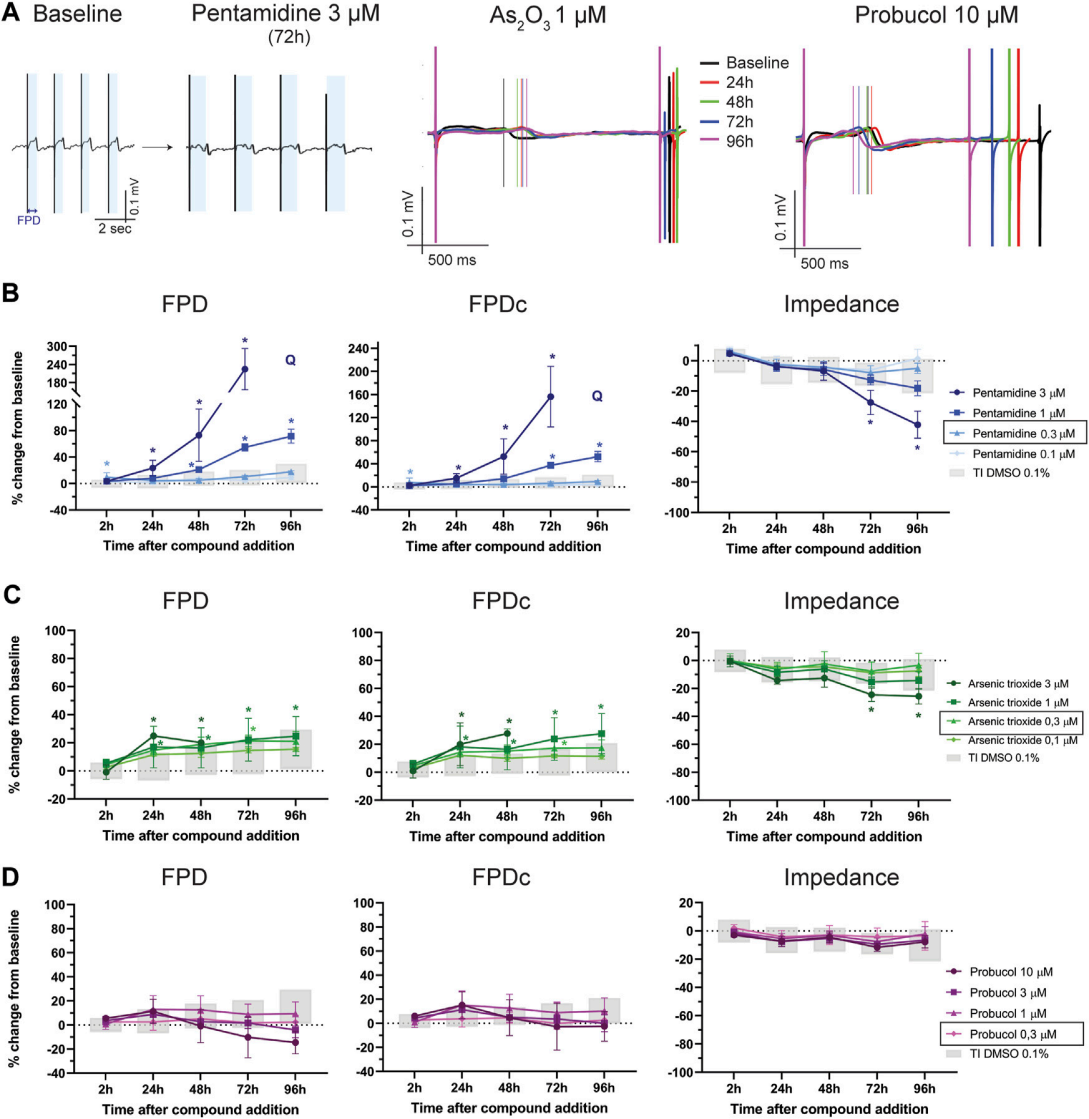
Ion-channels trafficking inhibition effect on FP. **(A)** Representative FP traces from baseline and after 72 h-treatment with pentamidine 3 µM. The light-blue shading indicates the FPD, significantly prolonged after treatment. In the center and right, representative FP traces at different time points of FP after treatment with As_2_O_3_ 1 µM and probucol 10 µM. The vertical lines indicate the top of the T-wave. Effect of **(B)** pentamidine, **(C)** As_2_O_3_ and **(D)** probucol on of FPD, FPDc and impedance. Q: quiescent (i.e., cessation of beating). In the figure legend, the doses indicated with a black outline are the closest to fC_max_. *: *p* < 0.05 and outside the tolerance intervals of vehicle controls.

One of the most important mechanisms of drug-induced cardiotoxicity is the impairment of cardiac ion channel trafficking. In the present study we tested three different compounds: pentamidine, arsenic trioxide and probucol. Pentamidine, an anti-protozoal drug, is known to induce QTc-prolongation and cause TdP in humans by interfering with the hERG channel trafficking (Cordes et al., 2005). We therefore aimed to understand the effect of a drug with this mechanism on FP. As depicted in Figures 5A, B, pentamidine induced a dose-dependent prolongation in FPD and FPDc (Supplementary Table S4). Interestingly, a physiologically relevant prolongation was observed in the 3 µM dose starting at 24 h and, for the 1 μM, 48 h after compound addition, consistent with the impairment of hERG trafficking previously described. Together with a dose-dependent decrease in BR (Supplementary Figure S3A), at the highest concentration of 3 μM, pentamidine caused cessation of beating in the hiPSC-CMs monolayer in 3/6 wells at 72 h and in 6/6 wells at 96 h, so the determination of FPD was not possible in the last time point (Supplementary Tables S4, S8). Furthermore, arrhythmic-like events were observed in 2/6 wells at 3 μM at 72 h timepoint (Supplementary Table S8).

Arsenic trioxide is often used to treat acute promyelocytic leukemia and has been linked to ventricular arrhythmias, sometimes resulting in sudden cardiac death (Vineetha and Raghu, 2019). As_2_O_3_ inhibits the maturation of hERG, thus leading to prolongation of the QTc interval and cardiac arrhythmias (Ficker et al., 2004). As shown in Figures 5A, C, As_2_O_3_ induced a prolongation of FPD that was more pronounced when corrected for beating rate (FPDc). At the highest dose (3 µM), the disappearance of the T-wave prevented the determination of the FPD at 72 and 96 h after compound addition. Interestingly, this concentration caused a significant reduction in impedance at 72 h and 96 h (Supplementary Tables S4, S8).

Probucol is a molecule used to lower LDL and HDL cholesterol, but cases of QTc prolongation and tachyarrhythmias have been associated to its administration (Cubeddu, 2016). Probucol impairs trafficking of hERG and K_V_7.1 (voltage-gated K^+^-channel 7.1), responsible for the rapid and slow component of the delayed-rectifier potassium current (*I*_Kr_ an *I*_Ks_), respectively, thus creating a link to the occurrence of QT prolongation and arrhythmias (Taniguchi et al., 2012). In hiPSC-CMs probucol did not induce a statistically significant prolongation of the FPD (Figures 5A, D) but caused a marked increase in BR (Supplementary Table S4 and Supplementary Figure S3) at the highest concentration (i.e., 10 µM). However, the FPD correction for beating rate did not show a physiologically relevant prolongation at any time points (Supplementary Table S8).

### 3.4 Effect of TKi on FPD and cytotoxicity

**FIGURE 6:**
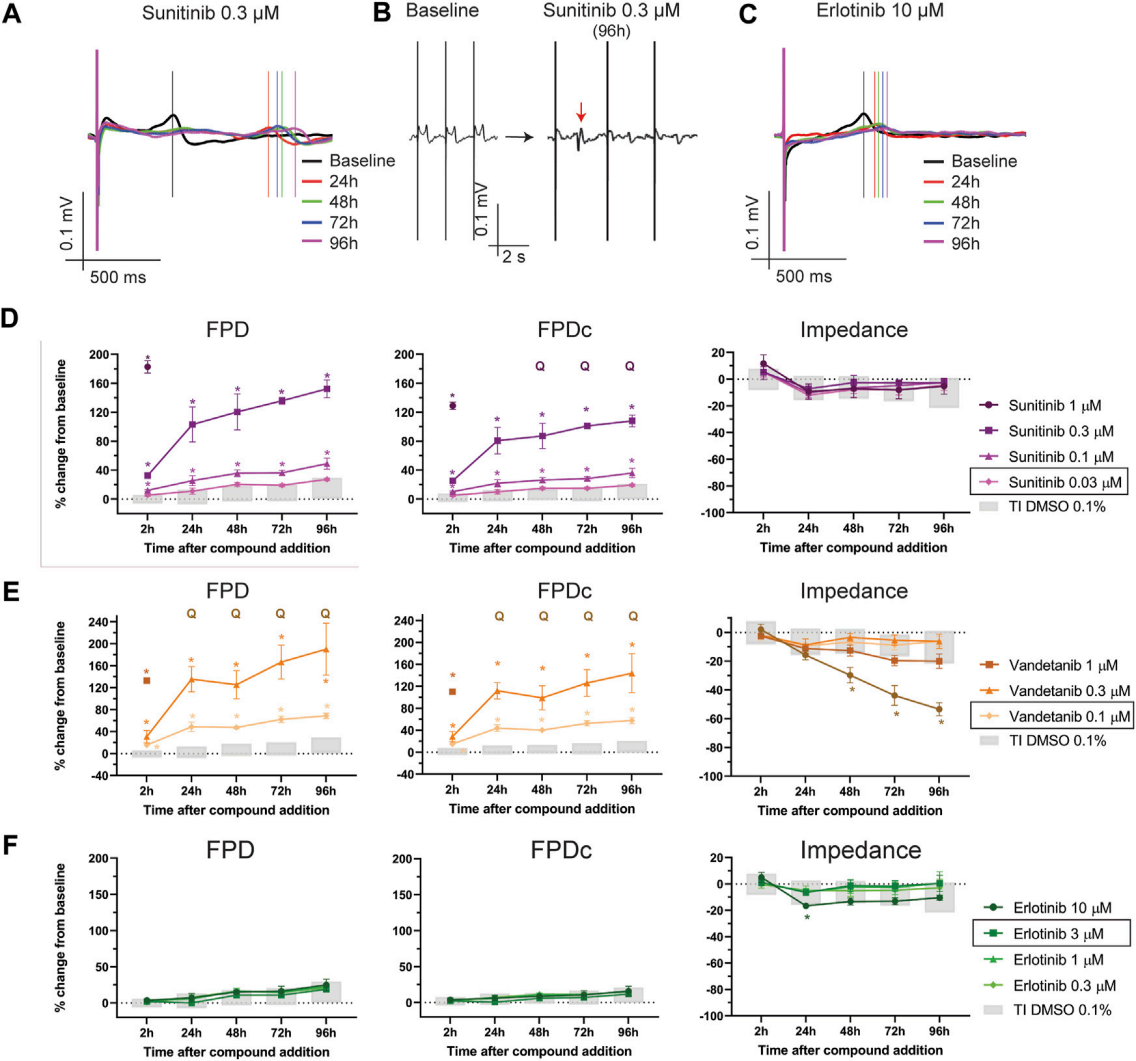
Effect of TKi on FP and impedance. **(A)** Representative traces depicting the time course of sunitinib 0.3 µM on FP. **(B)** Effect of sunitinib 0.3 µM on the morphology of the T-wave (the red arrow indicates an EADs-like event). **(C)** Representative traces depicting the time course of erlotinib 10 µM on FP. Effect of **(D)** sunitinib, **(E)** vandetanib and **(F)** erlotinib on FPD, FPDc. Q: quiescent (i.e., cessation of beating). The doses indicated in the figure legend with a black outline are the closest to fC_max_. *: *p* < 0.05 and outside the tolerance intervals of vehicle controls.

TK-inhibitors are a class of compounds used for the treatment of different types of cancer and several molecules from this class were associated with cardiotoxicity, such as sunitinib, vandetanib and nilotinib. Others, like erlotinib and axitinib, showed instead a safer profile (Orphanos et al., 2009). We sought to understand whether our assay was able to recapitulate such differences. Figure 6 shows the impact of TKi on FPD, FPDc and impedance. Sunitinib caused significant dose-dependent prolongation of the FPD at 0.3 and 0.1 µM, both at acute and chronic time points (Figures 6A, B, D, Supplementary Table S5). Sunitinib induced a concentration-dependent decrease in BR (Supplementary Figure S4) and, at the highest concentration tested (1 µM) the cessation of beating in all wells, starting at 24 h. Furthermore, EAD-*like* events occurred at chronic time points (starting at 24 h) for the 0.3 µM concentration (Supplementary Table S9). Interestingly, sunitinib did not cause significant changes in the impedance signal, suggesting a predominant electrophysiological component involved in cardiotoxicity at these concentrations (Figure 6D).

Vandetanib, also a high-risk profile TKi, caused similar effects as sunitinib, with marked concentration-dependent prolongation of FPD (and FPDc) at all concentrations tested, at both acute and chronic time points (Figure 6E, Supplementary Table S5, Supplementary Figure S4). EAD-*like* events occurred at the 3 µM concentration at 2 h, while, starting at 24 h, all wells turned quiescent (Supplementary Table S9). Cytotoxicity was also induced at this concentration, as shown in the impedance signal (Figure 6E, rightmost panel, Supplementary Table S5). Interestingly, the lowest concentration of 0.1 µM (4-times lower than the therapeutic fC_max_) induced a significant prolongation in the FPD (corrected and not corrected for beating rate), without any effect on impedance. Nilotinib, another high-risk profile TKi, induced a significant dose-dependent prolongation of the FPD at 1 and 0.3 µM, both at acute and chronic time points, without relevant changes to impedance (Supplementary Table S6). At 1 μM, one of five wells displayed EAD-like events but no cessation of beating occurred in any concentration (Supplementary Table S9, Supplementary Figure S4).

Erlotinib is considered to have a safer profile for cardiotoxicity (Orphanos et al., 2009). In this assay, we did not observe any physiologically relevant prolongation in FPD and FPDc (Figures 6C, F, Supplementary Table S6), nor significant changes in BR (Supplementary Figure S4). One well on 5, at the highest concentration (i.e., 10 µM) had a flat T-wave, but no arrhythmic-like events were observed (Supplementary Table S9). Interestingly, the highest concentration induced a borderline decrease in impedance, which was statistically and physiologically significant only at the 24 h time point.

### 3.5 Cytotoxicity assays and cell-death mechanisms

**FIGURE 7:**
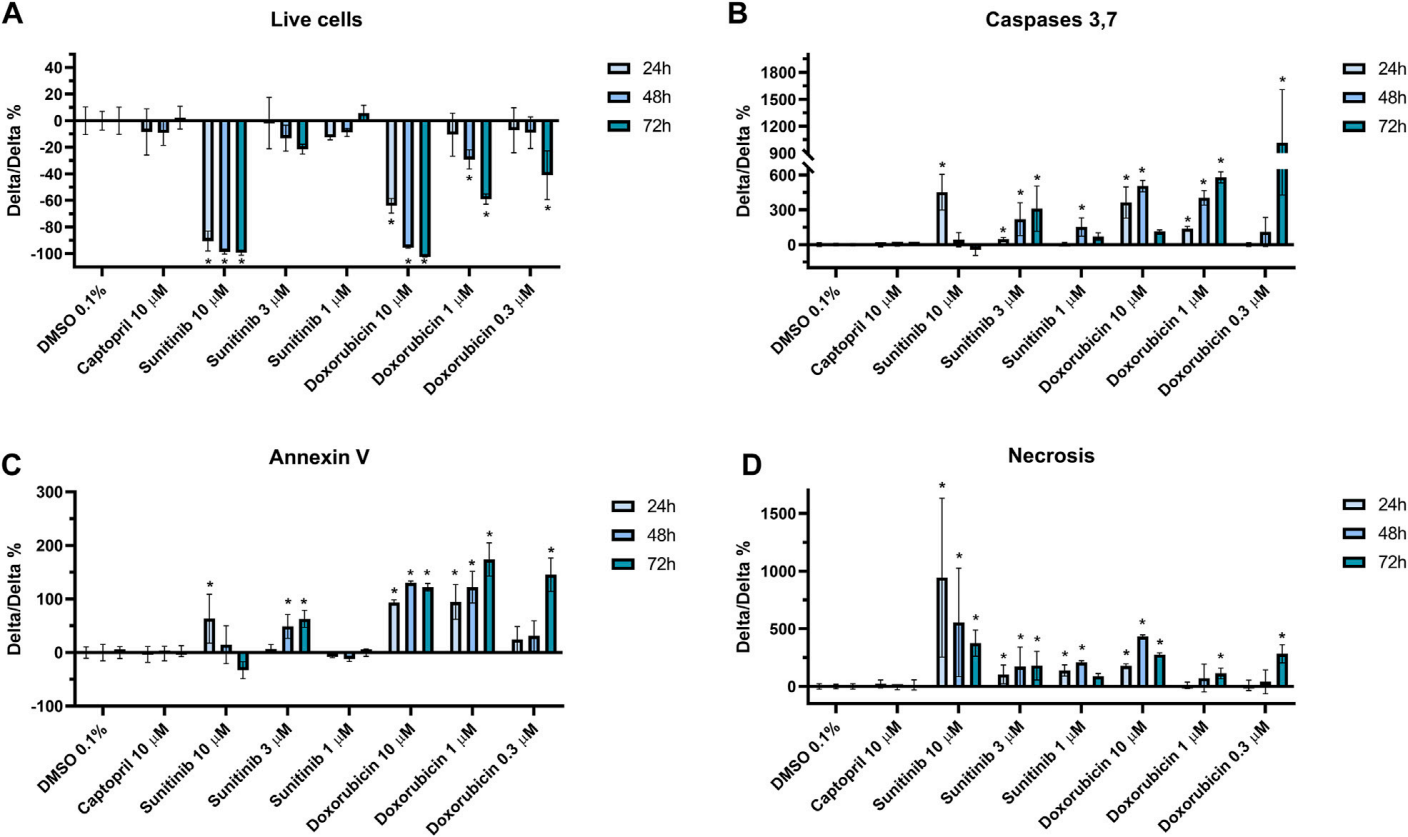
Cytotoxicity and cell-death mechanisms assays. Assays measuring the impact of the drug treatments on **(A)** cell viability, **(B)** apoptosis as reflected by caspases 3,7 or **(C)** Annexin V activation, and **(D)** necrosis. Data are normalized by vehicle control (DMSO 0.1%) and expressed as mean ± SD. “*” indicates *p* < 0.05.

In order to compare the results obtained using the functional assays, especially those from the impedance, we investigated the effect on apoptosis and necrosis of captopril (negative control), doxorubicin and sunitinib on hiPSC-CMs monolayers at 24, 48, and 72 h after compounds addition. Data are summarized in Supplementary Table S10. First, we looked at the number of viable cells as a measure of cytotoxicity. As shown in Figure 7A, sunitinib 10 µM induced a significant decrease in the number of live cells, while at 3 µM the compound did not cause a relevant change. Doxorubicin, at the highest concentration tested (i.e., 10 µM) also had a strong impact on the number of live cells, further decreasing over time. Similar effects, but with a lower magnitude, were detected when doxorubicin was tested at 1 and 0.3 µM. Captopril instead, did not show any significant change in live cell number at any of the time points. We then aimed to understand the mechanisms of cytotoxicity induced by these compounds. We therefore tested whether cell death was driven by apoptotic or necrotic pathways. As shown in Figures 7B, C, doxorubicin induced the largest activation of caspases 3,7, and of Annexin V, all indicating a predominant apoptosis activation. Over time, especially at 72 h, doxorubicin also showed the activation of necrosis (Figure 7D). Sunitinib instead seems to cause a predominant necrosis-driven cell death, especially at the high concentration of 10 µM. Captopril did not display any significant changes in necrosis or apoptosis activation.

## 4 Discussion

In the present study of *in vitro* assessment of chronic drug-induced cardiotoxicity, both functional electrophysiological readouts and cytotoxicity measurements were integrated at acute and chronic time points (up to 96 h) using a set of 13 well published clinical reference drugs. The study describes an *in vitro* approach using hiPSC-CMs to capture different mechanisms of drug-induced cardiotoxicity (including impaired ion-channel trafficking and cytotoxicity) from both electrical and impedance signals and confirmed by live-cell count, apoptosis and necrosis activation assays.

Drug-induced cardiotoxicity is associated with complex and diverse phenomena that can arise both from an impairment of cardiac electrophysiology and the direct loss of cardiac function (or a combination thereof). Traditionally, within the pharmaceutical industry, the primary focus of the risk assessment process is the evaluation of compounds effect on both ion channels *in vitro* and the QT interval of the ECG *in vivo*, as prolongation of the latter interval is known to potentially predispose to life-threatening arrhythmias. Currently, scrutiny of potential delayed drug-induced cardiac effects [which may even arise with unidentified modes of action with new therapeutic modalities, such as in the case of monoclonal antibodies (Dempsey et al., 2021)] is on the increase. It seems therefore important to assess compounds both acutely after dosing and at chronic time points. Preferably this is done at the earliest stages of drug development as possible, to avoid high development costs and potential risks in clinical studies.

In the present study, hiPSC-CMs were used in a functional *in-vitro* assay based on MEAs technology and the changes in the field-potential duration (FPD) and impedance over time, as a surrogate of the QT interval and, impedance, as an indicator of cytotoxicity were assessed up to 96 h. The interpretation of the effects of compounds vs. vehicle-treated wells and the choice of parameters to focus on was based on non-parametric tolerance intervals of vehicle experiments taken from 15 plates and using four different hiPSC-CMs batches.

The first example of a drug that induces delayed electrical changes is pentamidine, an antimicrobial compound used in the treatment of protozoal infections (especially in HIV patients). This drug was reported to induce QT prolongation, ventricular arrhythmias (including TdP) and sudden cardiac death in man (Girgis et al., 1997). The majority of the TdP cases occurred after prolonged intravenous administration of the drug, and only hours or days after the infusions (Eisenhauer et al., 1994), with exposures ranging from 1 to 5 µM (Sands et al., 1985; Conte et al., 1986; Cordes et al., 2005). In fact, *in vitro* studies revealed that pentamidine can reduce *I*_Kr_ density with an IC_50_ of 5–8 µM in heterologous expression systems, only after (at least) 24 h incubation, due to an inhibition of hERG channel trafficking (Cordes et al., 2005; Kuryshev et al., 2005). In this study, pentamidine induced a time- and dose-dependent prolongation of FPD and EAD-*like* events starting at 1 μM, in line with clinically reported exposures. A stronger effect on FPD was observed at 3 μM, a concentration that also induced cytotoxic effects at 48 h post compound addition, consistent with previously reported *in vitro* data (Cordes et al., 2005; Asahi et al., 2019). Interestingly, inhaled pentamidine (nasal spray formulations) exposures are often in the nM range and those have not been associated to either QT prolongation or TdP (Thalhammer et al., 1993). Correspondingly, we demonstrated that the lowest doses used in the study (i.e.,: 0.3–0.1 µM) did not result in a significant prolongation of the FPD.

Another example of a drug with similar delayed effects is arsenic trioxide (As_2_O_3_), which is used to treat acute promyelocytic leukemia. This compound has also been associated with cardiac arrhythmias and sudden cardiac death (Westervelt et al., 2001; Vineetha and Raghu, 2019). Like pentamidine, As_2_O_3_ impairs the trafficking of hERG thus explaining the link to life-threatening cardiac arrhythmias (Vineetha and Raghu, 2019). In our study, after 24 h treatment, As_2_O_3_ induced a prolongation in FPD, more pronounced in beat-rate corrected FPD, at relevant therapeutic concentrations. In fact, FPDc was significantly prolonged at 1 and 3 μM, consistent with a therapeutic fC_max_ of 0.3 µM. A mild cytotoxic effect, demonstrated by a significant reduction in impedance, was also observed at the highest concentration of 3 µM and at the latest timepoint.

A third reference drug we tested was probucol, which is used in the clinic to lower LDL and HDL cholesterol. This compound is also reported to interfere *in vitro* with hERG and K_V_7.1 channel trafficking (Taniguchi et al., 2012). However, in our study we did not observe a significant prolongation of FPD or FPDc, even at the highest concentration (i.e., 10 μM, 25-times the fC_max_). Although probucol is reported to reduce hERG expression in the plasma membrane without directly blocking hERG channel (Shi et al., 2015) and to prolong QT and cause TdPs in patients and in animal studies (Nogawa et al., 2013), the prolongation of the QT interval is not as evident as after treatment with other drugs, such as from arsenic trioxide and/or pentamidine. In men, Hayashi et al. (2004) reported the occurrence of TdP and excessive QT prolongation during a 3-month treatment with probucol in a long-QT syndrome patient carrying a missense hERG mutation. In an older study, authors suggested that probucol-induced lengthening of the QTc and subsequent arrhythmias occurrence is linked to several risk factors, such as female sex, longer QTc before treatment and low serum albumin level (Ohya et al., 1993). Taken together, it appears that QTc prolongation and TdP induced by probucol might be more evident only in patients with concomitant underlying risk factors (e.g., congenital long QT syndrome, female sex, *etc.*). An *in vitro* study using human embryonic stem cell-derived cardiomyocytes showed that 24 h-treatment with probucol (10 µM) only prolonged FPDc by ∼16%, compared to 4% in vehicle-treated wells, while in contrast large prolongation (62%) was observed with pentamidine (10 µM) (Hihara et al., 2014). From a mechanistic point of view, we can speculate that the increase in the beating rate and shortening of the FPD reported in our study might be caused by an effect of probucol on the composition of the membrane lipid content. It has been shown, in fact, that in rabbit ventricular myocytes, cholesterol contributes to the regulation of L-type Ca^2+^-current and its modulation by β-adrenergic signaling (Tsujikawa et al., 2008). In general, cholesterol plays a crucial role in the modulation of ion-channels membrane localization and function (Zakany et al., 2020), therefore the effect on hERG trafficking might have been concealed by a reorganization of ion-channels macromolecular complexes. Furthermore, as demonstrated by Taniguchi et al., probucol causes, additional to its effect on *I*_Kr_, an inhibition of *I*_Ks_-encoding K_V_7.1 channel trafficking (Taniguchi et al., 2012). In hiPSC-CMs, *I*_Ks_ only marginally contributes to the repolarization process (Zeng et al., 2019), thus the effect of probucol on repolarization prolongation due to the trafficking of *I*_Ks_ and *I*_Kr_ currents is more pronounced in freshly isolated cardiomyocytes or *in vivo*.

Taken together, the assay presented in the study was able to recapitulate the effect of two major hERG-trafficking inhibitors, pentamidine and arsenic trioxide, showing a prolongation of FPD and arrhythmic events, at relevant therapeutic exposures, but not probucol, which may be consistent with the mixed reports of trafficking effect in the literature.

In addition, as already mentioned, drug-induced cardiotoxicity can occur through loss of viable cardiomyocytes in the heart, leading to impaired cardiac function and heart failure. This is the case of for several classes of oncological compounds and antiviral drugs.

Doxorubicin is an effective chemotherapeutic agent used to treat different types of cancer (Octavia et al., 2012). Nevertheless, doxorubicin administration has been strongly correlated to heart failure in a significant portion of patients. Left-ventricular ejection fraction (LVEF) decreases in a dose-dependent manner, while congestive heart failure occurs in up to 48% of patients treated with doses of doxorubicin above 700 mg/m^2^ (Wenningmann et al., 2019). In the present study, we demonstrated that doxorubicin induced a concentration-dependent drop in the impedance signal, together with cessation of beating at therapeutic exposures. Furthermore, the cytotoxicity assays also confirmed a reduction in the live cells count, driven primarily by apoptosis, as shown by caspases 3 and 7 activation. Interestingly, doxorubicin 0.3 µM, at clinically relevant free plasma concentrations, induced a strong decrease in the impedance signal at 72 h (of ∼70%), while the live cells assay showed a drop of ∼40%. Interestingly (as summarized in Supplementary Figure S5), significant changes in all electrophysiological and cytotoxicity parameters (except for necrosis) occur at concentrations lower than fC_max_. This suggests that the impedance assay also detects changes in shape and morphology of the hiPSC-CMs monolayer, as well as cell detachment from the electrodes, as also demonstrated by others (Lei, 2014).

Another example of a compound with direct cardiotoxicity is BMS-986094, which was developed to treat hepatitis C infections. The compound was stopped after a phase II clinical trial in which several patients developed delayed alteration in the ECG and reduced LVEF within 6 months after administration of the compound, even leading to the death of one patient (Ahmad et al., 2015). In the present study, BMS-986094 induced changes in the FP morphology, including the flattening of the T-wave and shortening of FPD, compatible with the effect of this compound on calcium-transient amplitude reported earlier (Yanagida et al., 2021). We also detected a significant decrease in impedance at relevant concentrations, suggesting a cytotoxic effect of this compound on hiPSC-CMs. This is consistent with other *in vitro* data (Yanagida et al., 2021), with results in cynomolgus monkeys (Gill et al., 2017) and, most importantly, with pathological findings in patients involved in the phase II clinical trial (Ahmad et al., 2015). Due to the unavailability of exposures obtained in the phase II clinical trial, a comparison with the concentrations used in the present study is not possible. Nevertheless, an estimated dose of 3 µM caused cardiotoxicity in cynomolgus monkeys, in line with our results *in vitro* (Gill et al., 2017).

The third example of cardiotoxicity are a series of compounds all in the class of tyrosine-kinase inhibitors (TKi), which have been developed to treat several types of cancer (Huang et al., 2020). Toxicity can arise from on- and off-target effects, and not necessarily class-specific. In fact, clinical and *in vitro* studies demonstrated that some TKi are linked to severe cardiotoxicity, such as sunitinib, nilotinib and vandetanib (Orphanos et al., 2009). However, compounds of the class of TKis do not all have the same degree of toxicity and some clinical studies have shown that, for example, the TKi erlotinib has a safer profile (Brandão et al., 2022). In the present study we demonstrated that sunitinib, nilotinib and vandetanib have different cardiotoxic effects that included the generation of arrhythmic events, marked prolongation of the FPD and quiescence. Interestingly, while vandetanib induced a significant decrease in impedance at the highest concentration tested, sunitinib (up to 1 µM) did not show cytotoxic effects on the impedance readout, nor in the live cell count. In contrast, a profound effect on hiPSC-CMs viability (live cell count) was observed at 10 μM, mainly driven by necrosis pathway activation. As summarized in Supplementary Figure S5, significant changes in FPD, BR and FPDc occurred (for the 48 h time point) at 1 and 3-fold the therapeutic fC_max_ value (and within the minimum-maximum fC_max_ value reported), while a decrease in live-cell count only occurred at a concentration higher than 300-times the median fC_max_. In this assay we did not detect any significant effect on hiPSC-CMs electrophysiology or cytotoxicity after erlotinib incubation, even up to ∼ three times the estimated fC_max_, which is in agreement with clinical (Brandão et al., 2022) and preclinical data (Sharma et al., 2017).

The combined MEA assay presented in this study recapitulated several mechanisms of drug-induced cardiotoxicity. By digging into the molecular mechanisms of a selected set of compounds using cytotoxicity assays, we demonstrated that the functional impedance parameter predicts apoptosis- or necrosis-driven cell death and the morphological changes in hiPSC-CMs monolayers preceding cell death. This combined approach of electrophysiology and impedance offers the possibility to more comprehensively assessing the chronic effects of compounds, using a medium-throughput, human-based platform, suitable for further upscaling (up to 96-well MEAs plates are available). Furthermore, integration of the impedance measurement with MEA electrophysiological parameters allows for a deeper understanding of the mechanisms causing drug-induced cardiotoxicity, such as in the case of TKis presented in this study. In fact, sunitinib and vandetanib had a similar electrophysiological profile characterized by excessive FPD prolongation and arrhythmias, but only vandetanib induced cytotoxic effects. Similarly, doxorubicin caused cessation of beating and the disappearance of the T-wave, a phenomenon common to different mechanisms of action of drugs [such as hERG- or sodium-channel block (Blinova et al., 2018)]. The impedance readout allowed us to understand that cytotoxic effects were driving the changes in the FP, which was further corroborated by the live-cell count assay.

As cardiotoxicity is still one of the leading causes of drug withdrawal from clinical development (or in some cases even from the market), it is important to integrate a human-based chronic evaluation of compounds as early as possible in development, in order to predict potential effects on protein (e.g., ion channels) trafficking, drug-induced transcriptional modulations and cytotoxicity. Currently, the only opportunity to detect delayed cardiotoxicities is generally from long-term toxicology studies on animals. Often the design of such toxicity studies is suboptimal for sensitive QT-effect detection. Moreover, *ex-vivo/in vitro* cardiac tissues from animals have a limited acute physiological timespan within which to study the effects of a drug. Hence there are preclinical opportunities for sensitive *in vitro* human based assays to detect these effects and reduce animal use. In the future, integration of a chronic hiPSC-CMs assay with the classical approach to safety study from ICH E14/S7B (International Council for Harmonization) could contribute to a more timely detection of cardiac liabilities earlier in the drug development process, ultimately contributing to a reduction in development costs, impact of drug withdrawal and increase the level of safety for patients.

## 5 Limitations of the study

The present *in vitro* study used hiPSC-CMs derived from one single human donor and thus does not represent the genetic diversity of a population of humans and additionally does not mimic the complete *in vivo* cellular complexity of the 3D heart (including atrial/ventricular and other diversity, complex feedback/feedforward mechanisms involved in the cardiovascular functioning of the body). Furthermore, hiPSC-CMs cultured in a dish lack inflammatory and immune system interactions, which are also known to be linked to drug-induced cardiac toxicities in humans. In addition, compared to freshly isolated human cardiomyocytes, hiPSC-CM cells do have some electrophysiological and structural limitations. In fact, hiPSC-CMs beat spontaneously due to a reduction in the expression of Kir2.x channels, responsible for the inward rectifier potassium current *I*_K1_ (Goversen et al., 2018), higher expression of HCN “pacemaker” channels, as compared to adult human cardiomyocytes (Ma et al., 2011). From a structural point of view, the sarcomeric-structure of hiPSC-CMs does not fully resemble the longitudinally organized one of adult cardiomyocytes (Ma et al., 2011). Finally, the actual presence of compounds in the wells was not examined and hence may be somewhat lower than nominal (given the cell culture medium contains 10% bovine serum). Consequently, although there is debate about how to best calculate margins from *in vitro* to *in vivo*, this may affect the accuracy of the margin comparison between the *in vitro* nominal concentrations and clinical plasma fC_max_.

## 6 Conclusion

In the present study, we described and characterized a functional MEA assay using hiPSC-CMs evaluating the effect of vehicle (DMSO 0.1%) and 13 clinical reference compounds on field potential. By combining the assessment of electrophysiological parameters and cytotoxicity (through impedance measurement) we were able to capture different mechanisms of drug-induced cardiotoxicity, including impaired ion-channel trafficking and direct cytotoxicity, at relevant therapeutic concentrations. This chronic approach can complement acute (high throughput) assays for the prediction of proarrhythmic liabilities of developing compounds, used in for, e.g., the CiPA initiative (Blinova et al., 2018). The early deselection of compounds based on hazard identification of chronic cardiotoxicities will help to avoid late-stage detection in *vivo* studies or even in the clinic.

## Data Availability

The original contributions presented in the study are included in the article/Supplementary Material, further inquiries can be directed to the corresponding author.
