# Supplementary material for: Evaluation of chronic drug-induced electrophysiological and cytotoxic effects using human-induced pluripotent stem cell-derived cardiomyocytes (hiPSC-CMs)
Source: Front Pharmacol. 2023 Jul 10;14:1229960. doi: 10.3389/fphar.2023.1229960 (PMC10364322; doi:10.3389/fphar.2023.1229960)

Supplementary Material

Evaluation of chronic drug-induced electrophysiological and cytotoxic effects using human-induced pluripotent stem cell-derived cardiomyocytes (hiPSC-CMs)

Altrocchi C. ^1*^, Van Ammel K ^1^, Steemans M.^2^, Kreir M.^1^, Tekle F.^3^, Teisman A.^1^_,_ Gallacher D.J. ^1^, Lu H.R^1^.

# Supplementary Tables

**Table S1**: Effect of the negative controls captopril and aspirin on FP parameters. Data as % change from baseline are expressed as mean ± SD.

| **Captopril** | | | | | | | **Aspirin** | | | | | |
| --- | --- | --- | --- | --- | --- | --- | --- | --- | --- | --- | --- | --- |
| **Parameter** | **Concentration** | 2h | 24h | 48h | 72h | 96h | **Concentration** | 2h | 24h | 48h | 72h | 96h |
| **BR** | 30 µM | 2±3 | 1±3 | -7±1 | -9±2 | -16±2 | 100 µM | 2±3 | -2±3 | -7±2 | -10±3 | -16±4 |
|  | 10 µM | 2±2 | 1±3 | -7±3 | -10±1 | -17±1 | 30 µM | 3±2 | 0±4 | -8±2 | -9±3 | -18±4 |
|  | 3 µM | 2±2 | 1±3 | -7±2 | -9±1 | -18±2 | 10 µM | 3±2 | 2±3 | -8±3 | -9±2 | -17±3 |
|  | 1 µM | 4±4 | 1±5 | -7±4 | -10±1 | -15±4 | 3 µM | 2±2 | 1±2 | -7±1 | -10±2 | -18±1 |
| **FPD** | 30 µM | 3.4±1.3 | 7.4±4.1 | 13.9±3.1 | 15.6±2 | 23.3±2.4 | 100 µM | 0.2±2.9 | 7.2±3.6 | 13.4±3 | 17.6±2 | 23.9±1.7 |
|  | 10 µM | 0.3±1.7 | 5.3±3.7 | 13.1±5.3 | 14.8±5.3 | 23.4±6.5 | 30 µM | 0.9±1.8 | 6.7±3.9 | 13.5±5.1 | 15.4±5 | 23.3±4.7 |
|  | 3 µM | 0.3±1.9 | 4.3±0.8 | 10.9±2.8 | 12±0.8 | 20.9±1.6 | 10 µM | 4±7.9 | 5.8±4.5 | 12.2±6.3 | 14.6±6.7 | 23.3±7.8 |
|  | 1 µM | 0±2.5 | 5±5.9 | 11.5±3.8 | 16±2.7 | 22±4.6 | 3 µM | 2.5±1.6 | 6.2±3 | 13.2±2.8 | 16.3±2.2 | 25.2±3.5 |
| **FPDc** | 30 µM | 4±1.3 | 7.7±2.9 | 11.4±2.7 | 12.1±2.1 | 16±2.1 | 100 µM | 1.3±1.9 | 6.6±2.4 | 10.7±2.1 | 13.9±1.5 | 17±1.3 |
|  | 10 µM | 1±1.6 | 5.7±2.8 | 10.3±4.5 | 11.2±4.9 | 16±5.7 | 30 µM | 1.9±1.3 | 6.7±2.6 | 10.6±4.2 | 11.6±4.2 | 15.5±3.3 |
|  | 3 µM | 1.1±1.6 | 4.6±1.6 | 8.2±1.8 | 8.6±0.6 | 13.5±1.6 | 10 µM | 4.8±7.5 | 6.2±3.2 | 9.3±5.3 | 10.8±6 | 15.6±5.7 |
|  | 1 µM | 0.8±1.8 | 5.1±4.4 | 8.8±2.5 | 11.9±2.1 | 15.1±3 | 3 µM | 3.1±1 | 6.4±2.5 | 10±2.6 | 12.3±1.9 | 17.2±3.3 |
| **Imp** | 30 µM | 8±2 | -4±3 | -8±2 | 1±5 | -4±6 | 100 µM | 8±1 | -7±4 | -11±3 | -2±6 | -6±7 |
|  | 10 µM | 9±3 | -5±3 | -10±5 | -1±5 | -4±5 | 30 µM | 6±4 | -4±3 | -8±3 | -1±2 | -3±2 |
|  | 3 µM | 9±3 | -6±3 | -7±4 | 1±4 | -1±4 | 10 µM | 6±2 | -8±3 | -7±3 | -4±5 | -6±6 |
|  | 1 µM | 7±5 | -7±1 | -9±4 | -2±3 | -4±5 | 3 µM | 5±3 | -9±4 | -10±2 | -2±2 | -7±3 |

Amp FP: field potential amplitude; BR: beat rate; FPD: field-potential duration; FPDc: field-potential duration corrected by beat-rate; Imp: impedance. *p<0.05 (and out of vehicle TI)

**Table S2**: Effect of the negative controls amoxicillin and cetirizine on FP parameters. Data as % change from baseline are expressed as mean ± SD.

| **Amoxicillin** | | | | | | | **Cetirizine** | | | | | |
| --- | --- | --- | --- | --- | --- | --- | --- | --- | --- | --- | --- | --- |
|  | **Concentration** | 2h | 24h | 48h | 72h | 96h | **Concentration** | 2h | 24h | 48h | 72h | 96h |
| **BR** | 30 µM | 5±4 | 15±6 | 1±5 | -3±8 | -20±4* | 3 µM | 3±2 | 1±4 | -5±2 | -5±2 | -5±5 |
|  | 10 µM | 8±3 | 18±6* | 3±4 | -1±8 | -19±5* | 1 µM | 3±2 | 1±4 | -4±4 | -6±2 | -6±5 |
|  | 3 µM | 8±1 | 16±3 | 2±2 | -4±3 | -20±4* | 0.3 µM | 2±3 | 2±2 | -4±3 | -4±1 | -6±2 |
|  | 1 µM | 8±1 | 16±2 | 3±2 | -2±3 | -21±2* | 0.1 µM | 2±1 | 2±2 | -1±3 | 0±2 | -5±2 |
| **FPD** | 30 µM | -0.1±3.5 | -4.2±3.4 | 5.6±1.7 | 8.8±5.4 | 16.4±1.3 | 3 µM | 4.9±3.1 | 14.6±2.3 | 14.5±3.8 | 12.9±3.8 | 14.4±6.3 |
|  | 10 µM | 0.5±3.7 | -3.1±4.9 | 6.6±2.8 | 9.6±7.3 | 18.3±5.7 | 1 µM | 3.3±5.6 | 19.3±18.2 | 20.8±13.7 | 17.3±16.7 | 19±15.6 |
|  | 3 µM | 0.7±2.8 | -2.7±2.9 | 5.1±3.9 | 9.6±3.7 | 18.2±7.7 | 0.3 µM | 2.9±2.5 | 12.5±1.8 | 12.1±2.3 | 9.2±4.1 | 10.3±1.1 |
|  | 1 µM | -1±2.2 | -2.8±1.5 | 4.3±3 | 8.1±4.8 | 16.9±3 | 0.1 µM | 3.2±0.7 | 8.9±2.4 | 8.3±2.5 | 7.8±1.4 | 8.9±2.9 |
| **FPDc** | 30 µM | 1.6±2.8 | 0.5±2.7 | 6±2.2 | 7.7±3.8 | 7.9±2.1 | 3 µM | 6.1±3.3 | 15.2±2.8* | 12.5±3 | 11.2±3 | 12.1±5 |
|  | 10 µM | 3±3.1 | 2.2±3.9 | 7.4±2.1 | 8.9±4.3 | 10.4±4.4 | 1 µM | 4.5±5 | 19.6±16.4 | 19±15 | 15.4±17.1 | 16.5±16.6 |
|  | 3 µM | 3.1±2.9 | 2.3±2.9 | 5.7±3.9 | 8.4±2.9 | 9.6±6.7 | 0.3 µM | 3.8±2.3 | 13±1.9 | 10.4±2.2 | 7.7±3.7 | 8.2±1.1 |
|  | 1 µM | 1.6±2.2 | 2.1±1.1 | 5.3±2.8 | 7±3.6 | 8.1±2 | 0.1 µM | 4.1±0.9 | 9.7±1.6 | 8±2.9 | 7.6±1.5 | 7.1±3.5 |
| **Imp** | 30 µM | 2±3 | 2±4 | -1±3 | -6±4 | -4±5 | 3 µM | -3±2 | -7±4 | -3±6 | -8±7 | -6±7 |
|  | 10 µM | 2±3 | 7±6 | -2±3 | 1±4 | -1±6 | 1 µM | -4±2 | -7±5 | -4±7 | -5±6 | -4±9 |
|  | 3 µM | 2±3 | 7±6 | -3±6 | 1±4 | -2±6 | 0.3 µM | -3±3 | -8±2 | -5±4 | -5±6 | -5±6 |
|  | 1 µM | 2±4 | 9±7 | -2±4 | 4±3* | 0±8 | 0.1 µM | -4±4 | -8±1 | -7±2 | -7±3 | -7±4 |

Amp FP: field potential amplitude; BR: beat rate; FPD: field-potential duration; FPDc: field-potential duration corrected by beat-rate; Imp: impedance. *p<0.05 (and out of vehicle TI)

**Table S3:** Effect of doxorubicin and BMS-986094 on FP parameters. Data as % change from baseline are expressed as mean±SD.

|  | **Doxorubicin** | | | | | | **BMS-986094** | | | | | |
| --- | --- | --- | --- | --- | --- | --- | --- | --- | --- | --- | --- | --- |
| **Parameter** | **Concentration** | 2h | 24h | 48h | 72h | 96h | **Concentration** | 2h | 24h | 48h | 72h | 96h |
| **BR** | 3 µM | 1±2 | 41±36* | N/A | N/A | N/A | 10 µM | 4±8 | 30±4* | 24±6* | 32±14* | 29±0* |
|  | 1 µM | 3±2 | 68±21* | N/A | N/A | N/A | 3 µM | 7±1 | 20±3* | 13±3 | 12±6* | 20±12* |
|  | 0.3 µM | 2±2 | 24±8* | 40±8* | 39±0* | N/A | 1 µM | 6±2 | 17±5 | 12±3 | 17±6* | 14±8* |
|  | 0.1 µM | 7±3 | 41±12* | 26±8* | 22±6* | -7±13 | 0.3 µM | 5±3 | 8±3 | 14±5 | 22±3* | 30±11* |
| **FPD** | 3 µM | 3.9±2.4 | -15.8±3.8* | N/A | N/A | N/A | 10 µM | -5.7±2.3 | -24.2±3.7* | -10.6±0* | N/A | N/A |
|  | 1 µM | 2.8±3.4 | N/A | N/A | N/A | N/A | 3 µM | -3.6±1.9 | -17±4.9* | -0.5±0 | N/A | N/A |
|  | 0.3 µM | 2.2±0.8 | -21±2.9* | N/A | N/A | N/A | 1 µM | -3.6±2.3 | -10.7±2.3* | -5.8±3* | -7.5±0* | -7.9±0* |
|  | 0.1 µM | -0.9±3.5 | -18.2±8.6* | -24.4±9.8* | -25±8.7* | -11.7±0.4* | 0.3 µM | 0.5±1 | -5.3±4.1 | -12.4±4.7 | -17.3±3.6* | -20.4±2.9* |
| **FPDc** | 3 µM | 4.3±2.4 | -2.9±4.7* | N/A | N/A | N/A | 10 µM | -4.4±2.8* | -17.1±3.6* | -2.4±0* | N/A | N/A |
|  | 1 µM | 4.2±3 | N/A | N/A | N/A | N/A | 3 µM | -1.6±1.8 | -11.8±5.5* | 2.6±0 | N/A | N/A |
|  | 0.3 µM | 2.8±1.1 | -14.6±1.7* | N/A | N/A | N/A | 1 µM | -1.8±2.1 | -6±2.7 | -2.5±2.1* | -2.3±0 | -5.5±0* |
|  | 0.1 µM | 1.2±2.7 | -8.8±6.9* | -18.8±8.7* | -20.4±8.9* | -16.2±0.1* | 0.3 µM | 2.1±1.1 | -2.7±3.8 | -8.4±4.1 | -11.6±3.5* | -14.3±1.9* |
| **Imp** | 3 µM | -2±0 | -56±5* | -85±2* | -87±0* | -88±1* | 10 µM | 2±1 | 4±4 | 2±10* | -11±12 | -41±20* |
|  | 1 µM | -1±2 | -56±4* | -86±1* | -89±2* | -89±2* | 3 µM | 3±5 | 1±2 | -5±3 | -19±6* | -38±5* |
|  | 0.3 µM | -1±1 | -23±6* | -47±6* | -70±7* | -81±7* | 1 µM | -1±2 | -1±4 | -5±3 | -16±2 | -29±4 |
|  | 0.1 µM | 3±5 | -8±5 | -20±4 | -27±4 | -30±8 | 0.3 µM | -4±2 | -3±2 | -6±3 | -8±3 | -18±6 |

Amp FP: field potential amplitude; BR: beat rate; FPD: field-potential duration; FPDc: field-potential duration corrected by beat-rate; Imp: impedance. *p<0.05 (and out of vehicle TI). N/A: data not available due to cessation of beating or flat T-wave.

**Table S4:** Effect of pentamidine, arsenic trioxide and probucol on FP parameters. Data as % change from baseline are expressed as mean±SD.

|  | **Pentamidine** | | | | | | **Arsenic Trioxide** | | | | | | **Probucol** | | | | | |
| --- | --- | --- | --- | --- | --- | --- | --- | --- | --- | --- | --- | --- | --- | --- | --- | --- | --- | --- |
| **Parameter** | Concentration | 2h | 24h | 48h | 72h | 96h | Concentration | 2h | 24h | 48h | 72h | 96h | Concentration | 2h | 24h | 48h | 72h | 96h |
| **BR** | 3 µM | -2±3 | -18±8* | -30±8* | -54±5* | N/A | 3 µM | 6±2 | -8±39 | 23±2* | 28±2* | 19±11 | 10 µM | 1±2 | 10±3 | 17±3 | 26±7* | 48±6* |
|  | 1 µM | -3±3 | -7±3 | -16±3* | -30±2* | -30±5* | 1 µM | 1±1 | 3±4 | 0±2 | 4±4 | 7±4 | 3 µM | 2±4 | 7±0 | 7±2 | 6±4 | 14±5* |
|  | 0.3 µM | -4±4 | 0±3 | -2±4 | -11±4 | -20±5 | 0.3 µM | 0±2 | -2±1 | -10±2 | -11±3 | -6±5* | 1 µM | 0±1 | 6±1 | 0±2 | 0±5 | 2±3 |
|  | 0.1 µM | -1±2 | 2±2 | -4±3 | -6±3 | -13±3 | 0.1 µM | 1±1 | 1±3 | -6±1 | -7±3 | -10±2* | 0.3 µM | 0±1 | 4±2 | -1±1 | -6±3 | 0±7 |
| **FPD** | 3 µM | 3.6±1.9 | 23.4±11.8* | 73.1±39.4* | 224.5±68* | N/A | 3 µM | -0.8±5.2 | 25±2.2* | 20.1±0* | N/A | N/A | 10 µM | 5.5±2.1 | 11.3±10 | -0.8±13.7 | -10.2±17 | -14.5±9.4 |
|  | 1 µM | 3.6±1.6 | 8±1 | 21.1±3.4* | 54.7±5.4* | 71.5±10.4* | 1 µM | 5.4±2.9 | 17±14.7* | 16.5±14.2* | 22.2±15.2* | 24.7±13.9* | 3 µM | 3.7±3.5 | 8.9±1.5 | 2.8±2.5 | 1.7±4.6 | -4.1±6.5 |
|  | 0.3 µM | 9.7±6.7* | 4.6±3.3 | 4.9±2.6 | 10.5±2.1 | 17.7±1.7 | 0.3 µM | 4.6±3.6 | 14.9±3.1* | 18.9±5.2* | 21.3±4.1* | 21±7.1 | 1 µM | 0.8±4.5 | 13±11.4 | 12.6±11.7 | 8.9±8.5 | 9.2±9.9 |
|  | 0.1 µM | 2.6±1.2 | 2±3.4 | 7.3±1.7 | 4.9±2.5 | 9.2±3.1 | 0.1 µM | 2.7±3.3 | 11.5±0.9 | 12.6±2.8 | 14.6±1.7 | 15.5±1.3 | 0.3 µM | 2.5±1.9 | 2.5±6.9 | 5.1±4.5 | 1.8±4.6 | 2.4±5.7 |
| **FPDc** | 3 µM | 2.7±2.1 | 15.2±8 | 52.6±30.7* | 156.4±52.5* | N/A | 3 µM | 0.9±5 | 20.1±15.3* | 27.8±0* | N/A | N/A | 10 µM | 6±2.3 | 15.1±11 | 4.7±14.8 | -2.8±19.3 | -2.5±12.4 |
|  | 1 µM | 2.5±1.7 | 5.5±1.6 | 14.3±2.3* | 37.5±4.3* | 52.5±8.8* | 1 µM | 5.9±2.7 | 18.1±15* | 16.4±14.7* | 23.8±15.3* | 27.6±14.4* | 3 µM | 4.4±2.3 | 11.3±1.4 | 4.9±2.3 | 3.6±3.3 | 0.1±7 |
|  | 0.3 µM | 8±7.4 | 4.5±3.1 | 4.1±2 | 6.4±2.9 | 9.3±1.9 | 0.3 µM | 4.7±3.2 | 14.3±3.4* | 15.2±4.4* | 17.3±4.9* | 17.5±5.6 | 1 µM | 1.1±4.5 | 15.1±11.7 | 12.5±11.6 | 8.8±8.8 | 10.3±10.9 |
|  | 0.1 µM | 2.2±1.4 | 2.8±3.1 | 5.7±2 | 3±2.9 | 4.3±2.8 | 0.1 µM | 2.9±3.2 | 12.1±1.5 | 10±2.1 | 11.6±1.3 | 11.4±2 | 0.3 µM | 2.6±1.7 | 3.8±6.8 | 4.5±4.4 | -0.2±4.7 | 2.7±6.2 |
| **Imp** | 3 µM | 5±2 | -4±2 | -7±6 | -28±8* | -42±9* | 3 µM | -1±4 | -14±3 | -13±7 | -24±5* | -26±5* | 10 µM | -3±1 | -7±4 | -4±4 | -12±3 | -8±4 |
|  | 1 µM | 5±2 | -4±3 | -6±4 | -13±3 | -18±5 | 1 µM | -1±2 | -8±4 | -6±6 | -15±3 | -14±6 | 3 µM | -2±3 | -7±4 | -5±4 | -10±2 | -7±2 |
|  | 0.3 µM | 6±2 | -3±4 | -4±4 | -8±5 | -5±3 | 0.3 µM | 0±5 | -6±5 | -3±9 | -8±7 | -3±9 | 1 µM | -1±2 | -6±4 | -3±3 | -8±3 | -2±5 |
|  | 0.1 µM | 6±3 | -2±5 | -5±4 | -6±3 | 1±6 | 0.1 µM | 0±2 | -5±3 | -4±3 | -9±1 | -7±4 | 0.3 µM | 2±2 | -4±4 | -3±7* | -4±6 | -3±10 |

Amp FP: field potential amplitude; BR: beat rate; FPD: field-potential duration; FPDc: field-potential duration corrected by beat-rate; Imp: impedance. *p<0.05 and outside the DMSO 0.1% TI. N/A: data not available due to cessation of beating or flat T-wave.

**Table S5:** Effect of TKis (sunitinib, vandetanib) on FP parameters. Data as % change from baseline as expressed as mean ± SD.

| **Sunitinib** | | | | | | | **Vandetanib** | | | | | |
| --- | --- | --- | --- | --- | --- | --- | --- | --- | --- | --- | --- | --- |
| Parameter | Conc. | 2h | 24h | 48h | 72h | 96h | Conc. | 2h | 24h | 48h | 72h | 96h |
| BR | 1 µM | -49±4* | -67±3* | -68±4* | -70±0* | N/A | 3 µM | -58±4* | -71±1* | N/A | N/A | N/A |
|  | 0.3 µM | -15±2* | -29±5* | -39±4* | -38±1* | -44±2* | 1 µM | -28±5* | -59±5* | -65±2* | -68±2* | -68±0* |
|  | 0.1 µM | -5±1 | -8±4 | -19±2* | -17±2* | -24±1 | 0.3 µM | -5±4 | -29±5* | -31±2* | -40±3* | -43±6* |
|  | 0.03 µM | 0±2 | -2±3 | -13±2 | -10±2 | -18±1 | 0.1 µM | 0±4 | -9±5 | -14±2* | -17±2* | -19±3 |
| FPD | 1 µM | 182.8±8.5* | N/A | N/A | N/A | N/A | 3 µM | N/A | N/A | N/A | N/A | N/A |
|  | 0.3 µM | 32.5±1.7* | 103.1±24.2* | 120.5±24.7* | 135.9±4.7* | 152.4±12.3* | 1 µM | 132.8±0* | N/A | N/A | N/A | N/A |
|  | 0.1 µM | 12.2±1.2* | 25.6±6.7* | 35.8±4.6* | 36.3±3.3* | 49±7.7* | 0.3 µM | 30.8±11.2* | 135.5±23.1* | 125.5±25.7* | 166.6±30.8* | 190.2±47.3* |
|  | 0.03 µM | 5.5±1.5 | 11±4.2 | 20.5±2.7 | 19.2±2 | 27.4±1.8 | 0.1 µM | 15.6±3.3* | 48.8±8.4* | 47.5±2.1* | 62.4±5.7* | 68.8±4.9* |
| FPDc | 1 µM | 128.9±4.9* | N/A | N/A | N/A | N/A | 3 µM | N/A | N/A | N/A | N/A | N/A |
|  | 0.3 µM | 25.4±1.4* | 80.7±18.4* | 87.2±17.5* | 101±3.5* | 108.1±8* | 1 µM | 110.2±0* | N/A | N/A | N/A | N/A |
|  | 0.1 µM | 10.3±1* | 21.9±5* | 26.3±3.6* | 28.2±2.6* | 36.1±6.4* | 0.3 µM | 28.5±9.1* | 112±14.9* | 99.2±21.9* | 126.3±24.4* | 144.1±35.5* |
|  | 0.03 µM | 5.3±1.7 | 10.1±3.1 | 14.9±1.9* | 15±1.8 | 19.3±1.7 | 0.1 µM | 15.3±2.3* | 44.1±5.9* | 40.3±1.1* | 52.6±4.9* | 57.6±5.7* |
| Imp | 1 µM | 12±7 | -9±6 | -7±7 | -8±7 | -5±6 | 3 µM | 2±4 | -16±3 | -30±5* | -44±7* | -53±5* |
|  | 0.3 µM | 5±6 | -7±3 | -3±5 | -3±2 | -3±3 | 1 µM | -3±2 | -11±3 | -13±4 | -20±4* | -20±5 |
|  | 0.1 µM | 6±3 | -10±4 | -7±4 | -5±3 | -2±3 | 0.3 µM | -2±1 | -9±4 | -3±3 | -5±4 | -6±5 |
|  | 0.03 µM | 5±4 | -12±3 | -8±6 | -8±4 | -4±4 | 0.1 µM | -3±2 | -9±2 | -7±2 | -9±3 | -6±3 |

Amp FP: field potential amplitude; BR: beat rate; FPD: field-potential duration; FPDc: field-potential duration corrected by beat-rate; Imp: impedance. *p<0.05 and outside the DMSO 0.1% TI. N/A: data not available due to cessation of beating or flat T-wave.

**Table S6**: Effect of TKis (nilotinib and erlotinib) on FP parameters. Data as % change from baseline as expressed as mean ± SD.

|  | **Nilotinib** | | | | | | **Erlotinib** | | | | | |
| --- | --- | --- | --- | --- | --- | --- | --- | --- | --- | --- | --- | --- |
|  | **Concentration** | 2h | 24h | 48h | 72h | 96h | **Concentration** | 2h | 24h | 48h | 72h | 96h |
| **BR** | 1 µM | -15±5* | -42±3* | -37±3* | -44±3* | -43±3* | 10 µM | 1±1 | -1±7 | -15±4 | -13±4 | -19±2 |
|  | 0.3 µM | -4±1 | -17±2* | -13±1 | -19±3* | -23±2 | 3 µM | 1±2 | 2±3 | -13±3 | -10±2 | -18±2 |
|  | 0.1 µM | -1±1 | -8±3 | -8±4 | -11±4 | -18±4 | 1 µM | 1±1 | 0±5 | -13±3 | -9±2 | -16±2 |
|  | 0.01 µM | 1±2 | -1±3 | -3±2 | -8±2 | -14±3 | 0.3 µM | 3±3 | -1±5 | -12±1 | -8±2 | -14±1 |
| **FPD** | 1 µM | 80.6±28.8* | 264.1±51.3* | 209.3±40.8* | 260.2±58.2* | 208.7±49.5* | 10 µM | 3.5±0.9 | 7.2±5.8 | 15.4±2.7 | 16.4±6.7 | 25±8.1 |
|  | 0.3 µM | 22.4±5.4* | 61.6±9.1* | 46.7±15.8* | 59.3±17.6* | 57±11* | 3 µM | 2±3 | 0.1±4 | 11±2.4 | 10.9±3.3 | 18.9±1.9 |
|  | 0.1 µM | 8.9±9.8 | 19.8±3.9* | 15.4±2.6 | 17.3±1.9 | 19.6±3.2 | 1 µM | 3.4±1 | 5.5±5.8 | 16.6±3.6 | 14.7±3.9 | 22.4±3.5 |
|  | 0.01 µM | 3.9±3.8 | 6.4±4.8 | 12.9±12.2 | 10.1±2.4 | 13.9±2.5 | 0.3 µM | 0.6±0.9 | 7.8±3.3 | 16.2±1.3 | 14.6±3.1 | 21.2±1.9 |
| **FPDc** | 1 µM | 70.6±24.2* | 200.7±36.5* | 164.9±31.8* | 196.4±43* | 156.3±38.4* | 10 µM | 3.9±1.1 | 6.3±4.1 | 8.7±1.6 | 10.6±6 | 16±6.6 |
|  | 0.3 µM | 20.8±5.2* | 52.4±8.3* | 39.9±14.5* | 48.5±15* | 44.7±10.1* | 3 µM | 2.4±2.4 | 0.7±3 | 5.9±1.1 | 7.1±2.8 | 11.7±1.1 |
|  | 0.1 µM | 8.6±10.1 | 16.6±4.1* | 12.7±3 | 12.5±1.7 | 11.8±4.2 | 1 µM | 3.8±0.8 | 5.6±4 | 11.3±2.2 | 11±3.3 | 15.1±2.5 |
|  | 0.01 µM | 4.1±4 | 6.1±4.9 | 11.9±11.5 | 7.1±2.3 | 8.7±2.6 | 0.3 µM | 1.6±1.1 | 7.4±1.9 | 11.5±0.7 | 11.4±2.8 | 15.1±1.6 |
| **Imp** | 1 µM | 4±7 | -8±6 | 13±17* | 2±5* | 4±14 | 10 µM | 5±4 | -17±2* | -13±3 | -13±3 | -10±2 |
|  | 0.3 µM | -1±3 | -11±5 | 3±7 | -2±4 | -1±5 | 3 µM | 1±3 | -6±2 | -1±5 | -2±4 | 0±6 |
|  | 0.1 µM | -2±3 | -12±3 | 4±10 | -6±4 | -1±5 | 1 µM | 1±2 | -6±3 | -2±5 | -3±5 | 1±9 |
|  | 0.01 µM | -2±3 | -14±3 | -4±4 | -4±15 | -3±15 | 0.3 µM | 0±3 | -5±3 | -5±4 | -5±6 | -3±6 |

Amp FP: field potential amplitude; BR: beat rate; FPD: field-potential duration; FPDc: field-potential duration corrected by beat-rate; Imp: impedance. *p<0.05 and outside the DMSO 0.1% TI.

**Table S7:** Effect of doxorubicin and BMS-986094 on qualitative parameters. Data are expressed as number of observations/total well numbers.

|  | **Doxorubicin** | | | | | | **BMS-986094** | | | | | |
| --- | --- | --- | --- | --- | --- | --- | --- | --- | --- | --- | --- | --- |
| **Parameter** |  |  |  |  |  |  |  |  |  |  |  |  |
|  | Concentration | 2h | 24h | 48h | 72h | 96h | Concentration | 2h | 24h | 48h | 72h | 96h |
| **Beating below threshold** | 3 µM | 0/5 | 0/5 | 0/5 | 0/5 | 0/5 | 10 µM | 0/5 | 0/5 | 0/5 | 0/5 | 1/5 |
|  | 1 µM | 0/5 | 0/5 | 0/5 | 0/5 | 0/5 | 3 µM | 0/5 | 0/5 | 0/5 | 0/5 | 0/5 |
|  | 0.3 µM | 0/5 | 0/5 | 0/5 | 0/5 | 0/5 | 1 µM | 0/5 | 0/5 | 0/5 | 0/5 | 0/5 |
|  | 0.1 µM | 0/5 | 0/5 | 0/5 | 0/5 | 0/5 | 0.3 µM | 0/5 | 0/5 | 0/5 | 0/5 | 0/5 |
| **EAD** | 3 µM | 0/5 | 0/5 | 0/5 | 0/5 | 0/5 | 10 µM | 0/5 | 0/5 | 0/5 | 0/5 | 0/5 |
|  | 1 µM | 0/5 | 0/5 | 0/5 | 0/5 | 0/5 | 3 µM | 0/5 | 0/5 | 0/5 | 0/5 | 0/5 |
|  | 0.3 µM | 0/5 | 0/5 | 0/5 | 0/5 | 0/5 | 1 µM | 0/5 | 0/5 | 0/5 | 0/5 | 0/5 |
|  | 0.1 µM | 0/5 | 0/5 | 0/5 | 0/5 | 0/5 | 0.3 µM | 0/5 | 0/5 | 0/5 | 0/5 | 0/5 |
| **Flat T-wave-like** | 3 µM | 0/5 | 2/5 | 0/5 | 0/5 | 0/5 | 10 µM | 0/5 | 1/5 | 4/5 | 5/5 | 1/5 |
|  | 1 µM | 0/5 | 4/5 | 0/5 | 0/5 | 0/5 | 3 µM | 0/5 | 0/5 | 4/5 | 5/5 | 4/5 |
|  | 0.3 µM | 0/5 | 2/5 | 3/5 | 1/5 | 0/5 | 1 µM | 1/5 | 0/5 | 3/5 | 4/5 | 4/5 |
|  | 0.1 µM | 0/5 | 0/5 | 0/5 | 1/5 | 3/5 | 0.3 µM | 0/5 | 0/5 | 0/5 | 0/5 | 1/5 |
| **Cessation of beating** | 3 µM | 0/5 | 1/5 | 5/5 | 5/5 | 5/5 | 10 µM | 0/5 | 0/5 | 0/5 | 0/5 | 3/5 |
|  | 1 µM | 0/5 | 0/5 | 5/5 | 5/5 | 5/5 | 3 µM | 0/5 | 0/5 | 0/5 | 0/5 | 1/5 |
|  | 0.3 µM | 0/5 | 0/5 | 2/5 | 4/5 | 5/5 | 1 µM | 0/5 | 0/5 | 0/5 | 0/5 | 0/5 |
|  | 0.1 µM | 0/5 | 0/5 | 0/5 | 0/5 | 0/5 | 0.3 µM | 0/5 | 0/5 | 0/5 | 0/5 | 0/5 |

**Table S8:** Effect of pentamidine, arsenic trioxide and probucol on qualitative parameters. Data are expressed as number of observations/total well numbers.

|  | **Pentamidine** | | | | | | **Arsenic Trioxide** | | | | | | **Probucol** | | | | | |
| --- | --- | --- | --- | --- | --- | --- | --- | --- | --- | --- | --- | --- | --- | --- | --- | --- | --- | --- |
| **Parameter** |  |  |  |  |  |  |  |  |  |  |  |  |  |  |  |  |  |  |
|  |  | 2h | 24h | 48h | 72h | 96h |  | 2h | 24h | 48h | 72h | 96h |  | 2h | 24h | 48h | 72h | 96h |
| **Beating below threshold** | 3 µM | 0/6 | 0/6 | 0/6 | 0/6 | 1/6 | 3 µM | 0/5 | 0/5 | 0/5 | 0/5 | 0/5 | 10 µM | 0/5 | 0/5 | 0/5 | 0/5 | 0/5 |
|  | 1 µM | 0/6 | 0/6 | 0/6 | 0/6 | 0/6 | 1 µM | 0/5 | 0/5 | 0/5 | 0/5 | 0/5 | 3 µM | 0/5 | 0/5 | 0/5 | 0/5 | 0/5 |
|  | 0.3 µM | 0/5 | 0/5 | 0/5 | 0/5 | 0/5 | 0.3 µM | 0/5 | 0/5 | 0/5 | 0/5 | 0/5 | 1 µM | 0/4 | 0/4 | 0/4 | 0/4 | 0/4 |
|  | 0.1 µM | 0/6 | 0/6 | 0/6 | 0/6 | 0/6 | 0.1 µM | 0/5 | 0/5 | 0/5 | 0/5 | 0/5 | 0.3 µM | 0/5 | 0/5 | 0/5 | 0/5 | 0/5 |
| **EAD** | 3 µM | 0/6 | 0/6 | 0/6 | 2/6 | 0/6 | 3 µM | 0/5 | 0/5 | 0/5 | 0/5 | 0/5 | 10 µM | 0/5 | 0/5 | 0/5 | 0/5 | 0/5 |
|  | 1 µM | 0/6 | 0/6 | 0/6 | 0/6 | 0/6 | 1 µM | 0/5 | 0/5 | 0/5 | 0/5 | 0/5 | 3 µM | 0/5 | 0/5 | 0/5 | 0/5 | 0/5 |
|  | 0.3 µM | 0/5 | 0/5 | 0/5 | 0/5 | 0/5 | 0.3 µM | 0/5 | 0/5 | 0/5 | 0/5 | 0/5 | 1 µM | 0/4 | 0/4 | 0/4 | 0/4 | 0/4 |
|  | 0.1 µM | 0/6 | 0/6 | 0/6 | 0/6 | 0/6 | 0.1 µM | 0/5 | 0/5 | 0/5 | 0/5 | 0/5 | 0.3 µM | 0/5 | 0/5 | 0/5 | 0/5 | 0/5 |
| **Flat T-wave-like** | 3 µM | 0/6 | 0/6 | 0/6 | 1/6 | 0/6 | 3 µM | 0/5 | 2/5 | 0/5 | 0/5 | 0/5 | 10 µM | 0/5 | 0/5 | 1/5 | 1/5 | 2/5 |
|  | 1 µM | 0/6 | 0/6 | 0/6 | 0/6 | 0/6 | 1 µM | 0/5 | 4/5 | 0/5 | 0/5 | 0/5 | 3 µM | 0/5 | 0/5 | 0/5 | 0/5 | 0/5 |
|  | 0.3 µM | 0/5 | 0/5 | 0/5 | 0/5 | 0/5 | 0.3 µM | 0/5 | 2/5 | 3/5 | 1/5 | 0/5 | 1 µM | 0/4 | 0/4 | 0/4 | 0/4 | 0/4 |
|  | 0.1 µM | 0/6 | 0/6 | 0/6 | 0/6 | 0/6 | 0.1 µM | 0/5 | 0/5 | 0/5 | 1/5 | 3/5 | 0.3 µM | 0/5 | 1/5 | 0/5 | 0/5 | 0/5 |
| **Cessation of beating** | 3 µM | 0/6 | 0/6 | 0/6 | 3/6 | 5/6 | 3 µM | 0/5 | 1/5 | 5/5 | 5/5 | 5/5 | 10 µM | 0/5 | 0/5 | 0/5 | 0/5 | 0/5 |
|  | 1 µM | 0/6 | 0/6 | 0/6 | 0/6 | 0/6 | 1 µM | 0/5 | 0/5 | 5/5 | 5/5 | 5/5 | 3 µM | 0/5 | 0/5 | 0/5 | 0/5 | 0/5 |
|  | 0.3 µM | 0/5 | 0/5 | 0/5 | 0/5 | 0/5 | 0.3 µM | 0/5 | 0/5 | 2/5 | 4/5 | 5/5 | 1 µM | 0/4 | 0/4 | 0/4 | 0/4 | 0/4 |
|  | 0.1 µM | 0/6 | 0/6 | 0/6 | 0/6 | 0/6 | 0.1 µM | 0/5 | 0/5 | 0/5 | 0/5 | 0/5 | 0.3 µM | 0/5 | 0/5 | 0/5 | 0/5 | 0/5 |

**Table S9:** Effect of TKis (sunitinib, vandetanib, nilotinib and erlotinib) on qualitative parameters. Data are expressed as number of observations/total well numbers.

|  | **Sunitinib** | | | | | | **Vandetanib** | | | | | | **Nilotinib** | | | | | | **Erlotinib** | | | | | |
| --- | --- | --- | --- | --- | --- | --- | --- | --- | --- | --- | --- | --- | --- | --- | --- | --- | --- | --- | --- | --- | --- | --- | --- | --- |
| **Parameter** |  |  |  |  |  |  |  |  |  |  |  |  |  |  |  |  |  |  |  |  |  |  |  |  |
|  | Conc. | 2h | 24h | 48h | 72h | 96h | Conc. | 2h | 24h | 48h | 72h | 96h | Conc. | 2h | 24h | 48h | 72h | 96h | Conc. | 2h | 24h | 48h | 72h | 96h |
| **Beating below threshold** | 1 µM | 0/5 | 0/5 | 0/5 | 0/5 | 0/5 | 3 µM | 0/5 | 0/5 | 1/5 | 0/5 | 0/5 | 1 µM | 0/5 | 0/5 | 0/5 | 0/5 | 0/5 | 10 µM | 0/5 | 0/5 | 0/5 | 0/5 | 0/5 |
|  | 0.3 µM | 0/5 | 0/5 | 0/5 | 0/5 | 0/5 | 1 µM | 0/5 | 0/5 | 0/5 | 1/5 | 0/5 | 0.3 µM | 0/5 | 0/5 | 0/5 | 0/5 | 0/5 | 3 µM | 0/5 | 0/5 | 0/5 | 0/5 | 0/5 |
|  | 0.1 µM | 0/5 | 0/5 | 0/5 | 0/5 | 0/5 | 0.3 µM | 0/5 | 0/5 | 0/5 | 0/5 | 0/5 | 0.1 µM | 0/5 | 0/5 | 0/5 | 0/5 | 0/5 | 1 µM | 0/5 | 0/5 | 0/5 | 0/5 | 0/5 |
|  | 0.03 µM | 0/5 | 0/5 | 0/5 | 0/5 | 0/5 | 0.1 µM | 0/5 | 0/5 | 0/5 | 0/5 | 0/5 | 0.01 µM | 0/5 | 0/5 | 0/5 | 0/5 | 0/5 | 0.3 µM | 0/5 | 0/5 | 0/5 | 0/5 | 0/5 |
| **EAD** | 1 µM | 0/5 | 0/5 | 0/5 | 0/5 | 0/5 | 3 µM | 0/5 | 0/5 | 0/5 | 0/5 | 0/5 | 1 µM | 1/5 | 1/5 | 1/5 | 1/5 | 1/5 | 10 µM | 0/5 | 0/5 | 0/5 | 0/5 | 0/5 |
|  | 0.3 µM | 0/5 | 2/5 | 3/5 | 0/5 | 1/5 | 1 µM | 0/5 | 1/5 | 2/5 | 0/5 | 0/5 | 0.3 µM | 0/5 | 0/5 | 0/5 | 0/5 | 0/5 | 3 µM | 0/5 | 0/5 | 0/5 | 0/5 | 0/5 |
|  | 0.1 µM | 0/5 | 0/5 | 0/5 | 0/5 | 0/5 | 0.3 µM | 0/5 | 1/5 | 0/5 | 0/5 | 0/5 | 0.1 µM | 0/5 | 0/5 | 0/5 | 0/5 | 0/5 | 1 µM | 0/5 | 0/5 | 0/5 | 0/5 | 0/5 |
|  | 0.03 µM | 0/5 | 0/5 | 0/5 | 0/5 | 0/5 | 0.1 µM | 0/5 | 0/5 | 0/5 | 0/5 | 0/5 | 0.01 µM | 0/5 | 0/5 | 0/5 | 0/5 | 0/5 | 0.3 µM | 0/5 | 0/5 | 0/5 | 0/5 | 0/5 |
| **Flat T-wave-like** | 1 µM | 2/5 | 5/5 | 2/5 | 1/5 | 0/5 | 3 µM | 5/5 | 2/5 | 0/5 | 0/5 | 0/5 | 1 µM | 0/5 | 2/5 | 0/4 | 1/5 | 1/5 | 10 µM | 0/5 | 1/5 | 1/5 | 1/5 | 1/5 |
|  | 0.3 µM | 0/5 | 0/5 | 0/5 | 0/5 | 0/5 | 1 µM | 4/5 | 5/5 | 5/5 | 3/5 | 1/5 | 0.3 µM | 0/5 | 0/5 | 0/5 | 0/5 | 1/5 | 3 µM | 0/5 | 0/5 | 0/5 | 0/5 | 0/5 |
|  | 0.1 µM | 0/5 | 0/5 | 0/5 | 0/5 | 0/5 | 0.3 µM | 0/5 | 1/5 | 1/5 | 1/5 | 1/5 | 0.1 µM | 0/5 | 0/5 | 0/5 | 0/5 | 0/5 | 1 µM | 0/5 | 0/5 | 0/5 | 0/5 | 0/5 |
|  | 0.03 µM | 0/5 | 0/5 | 0/5 | 0/5 | 0/5 | 0.1 µM | 0/5 | 0/5 | 0/5 | 0/5 | 0/5 | 0.01 µM | 0/5 | 0/5 | 0/5 | 0/5 | 0/5 | 0.3 µM | 0/5 | 0/5 | 0/5 | 0/5 | 0/5 |
| **Cessation of beating** | 1 µM | 0/5 | 0/5 | 3/5 | 4/5 | 5/5 | 3 µM | 0/5 | 3/5 | 4/5 | 5/5 | 5/5 | 1 µM | 0/5 | 0/5 | 0/5 | 0/5 | 0/5 | 10 µM | 0/5 | 0/5 | 0/5 | 0/5 | 0/5 |
|  | 0.3 µM | 0/5 | 0/5 | 0/5 | 0/5 | 0/5 | 1 µM | 0/5 | 0/5 | 0/5 | 1/5 | 4/5 | 0.3 µM | 0/5 | 0/5 | 0/5 | 0/5 | 0/5 | 3 µM | 0/5 | 0/5 | 0/5 | 0/5 | 0/5 |
|  | 0.1 µM | 0/5 | 0/5 | 0/5 | 0/5 | 0/5 | 0.3 µM | 0/5 | 0/5 | 0/5 | 0/5 | 0/5 | 0.1 µM | 0/5 | 0/5 | 0/5 | 0/5 | 0/5 | 1 µM | 0/5 | 0/5 | 0/5 | 0/5 | 0/5 |
|  | 0.03 µM | 0/5 | 0/5 | 0/5 | 0/5 | 0/5 | 0.1 µM | 0/5 | 0/5 | 0/5 | 0/5 | 0/5 | 0.01 µM | 0/5 | 0/5 | 0/5 | 0/5 | 0/5 | 0.3 µM | 0/5 | 0/5 | 0/5 | 0/5 | 0/5 |

**Table S10:** Effect of captopril, sunitinib and doxorubicin on viability (live cells), apoptosis and necrosis activation measured by fluorescence- and luminescence-based plate reader assays. Data as Δ/Δ% are summarized as mean, SD and number of replicates (N) per concentration, at each time point analyzed (24, 48 and 72h).

|  | **Live Cells** | | | | | | **Caspases 3, 7** | | | | | |
| --- | --- | --- | --- | --- | --- | --- | --- | --- | --- | --- | --- | --- |
|  | 24h | | 48h | | 72h | | 24h | | 48h | | 72h | |
| **Compound** | Mean±SD | N | Mean±SD | N | Mean±SD | N | Mean±SD | N | Mean±SD | N | Mean±SD | N |
| **DMSO 0.1%** | 0±10.3 | 12 | 0±7 | 12 | 0±10.2 | 12 | 0±16.2 | 12 | 0±11.4 | 12 | 0±10.5 | 12 |
| **Captopril 10 μM** | -8.4±17.4 | 8 | -9.1±9.5 | 8 | 2.4±8.5 | 8 | -6.9±11.1 | 8 | -3±10.7 | 8 | -3.6±5 | 8 |
| **Sunitinib 10 μM** | -90.4*±7.4 | 8 | -98.6*±1.8 | 8 | -99.3*±1.9 | 8 | 451.6*±153.6 | 8 | 42.9±61.5 | 8 | -45±134.8 | 8 |
| **Sunitinib 3 μM** | -1.8±19.3 | 8 | -13.1±9.7 | 8 | -21.4±3.7 | 8 | 49.5*±13 | 8 | 219.7*±141.3 | 8 | 310.5*±271.2 | 8 |
| **Sunitinib 1 μM** | -12.5±1.8 | 4 | -8.8±2.9 | 4 | 5.8±5.9 | 4 | 11±8.3 | 4 | 152.7*±78.7 | 4 | 70.2±31.9 | 4 |
| **Doxorubicin 10 μM** | -64*±5.5 | 4 | -95.5*±0.4 | 4 | -102.4*±0.2 | 4 | 363.8*±133.5 | 4 | 504.6*±49.8 | 4 | 113±14.3 | 4 |
| **Doxorubicin 1 μM** | -10.5±16.2 | 8 | -29.2*±7.2 | 8 | -59*±3.8 | 8 | 137.3*±20.7 | 8 | 403.6*±62.2 | 8 | 580.1*±48.2 | 8 |
| **Doxorubicin 0.3 μM** | -7.1±16.9 | 8 | -9±11.9 | 8 | -41*±18.3 | 8 | 0.6±15.9 | 8 | 109.5±125.1 | 8 | 1017.3*±590.8 | 8 |
|  | **Annexin V** | | | | | | **Necrosis** | | | | | |
|  | 24h | | 48h | | 72h | | 24h | | 48h | | 72h | |
|  | Mean±SD | N | Mean±SD | N | Mean±SD | N | Mean±SD | N | Mean±SD | N | Mean±SD | N |
| **DMSO 0.1%** | 0±10.6 | 12 | 0±15.6 | 12 | 0±11.1 | 12 | 0±22.7 | 12 | 0±19.4 | 12 | 0±23.1 | 12 |
| **Captopril 10 μM** | -3.4±14.7 | 8 | -1.8±13.6 | 8 | 2.6±10.3 | 8 | 21.5±33.7 | 8 | -8.1±21.1 | 8 | 13.9±44.2 | 8 |
| **Sunitinib 10 μM** | 63.3*±45.6 | 8 | 14.8±35.2 | 8 | -32.7±15.7 | 8 | 943.5*±688.2 | 7 | 555.2*±470.7 | 7 | 375.6*±113.1 | 8 |
| **Sunitinib 3 μM** | 7±8.3 | 8 | 48.9*±22.2 | 8 | 63*±15.8 | 8 | 104.3*±81.4 | 8 | 172.1*±168.3 | 8 | 179.9*±123.7 | 8 |
| **Sunitinib 1 μM** | -7.5±2.1 | 4 | -11.9±4.5 | 4 | -0.3±7 | 4 | 138.4*±48.2 | 4 | 208.3*±14.2 | 4 | 88.5±23.2 | 4 |
| **Doxorubicin 10 μM** | 93.6*±5.1 | 4 | 130.2*±3.8 | 4 | 122.1*±7.2 | 4 | 177.2*±19 | 4 | 434.1*±13.3 | 4 | 276*±15.7 | 4 |
| **Doxorubicin 1 μM** | 94.6*±32.5 | 8 | 122.2*±29.8 | 8 | 173.8*±31.1 | 8 | 9.8±27 | 8 | 72.9±120.4 | 8 | 112.9*±45.7 | 8 |
| **Doxorubicin 0.3 μM** | 24.6±24 | 8 | 31.2±28.5 | 8 | 145.3*±31 | 8 | 9.8±45.2 | 8 | 40.2±103.1 | 8 | 282.3*±78.1 | 8 |

*: p<0.05 vs. DMSO 0.1%

**Supplementary Figure S1:** **Effect of negative controls on beating rate.** Electrophysiological changes in BR (as mean±SD) caused by **A)** captopril, **B**) amoxicillin, **C)** cetirizine and **D)** aspirin. In the figure legend, the doses indicated with a black outline are the closest to fC_max_. *: p<0.05 and outside the tolerance intervals of vehicle controls.


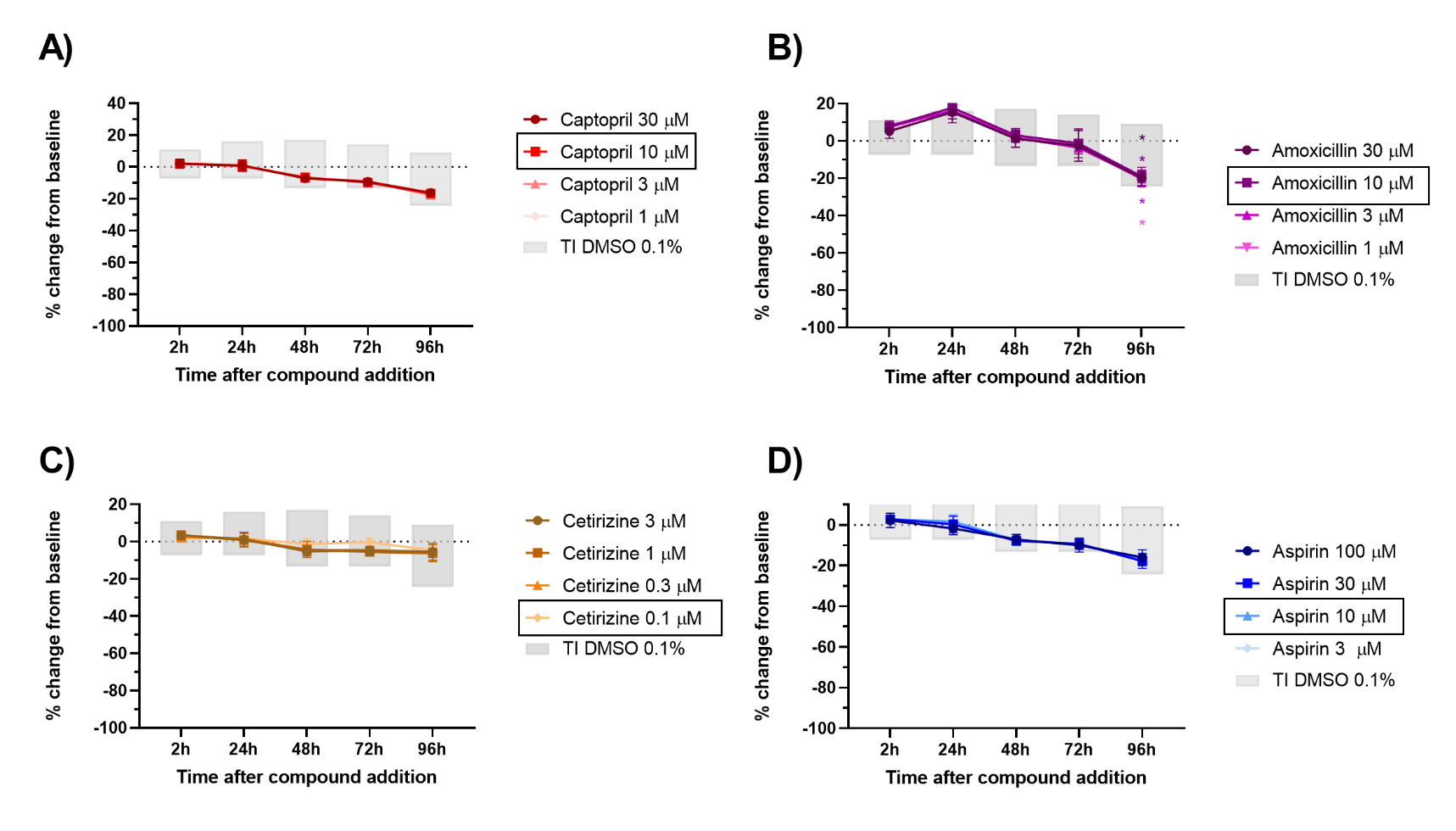


**Supplementary Figure S2:** **Effect of doxorubicin and BMS-986094 on beating rate.** Electrophysiological changes in BR (as mean±SD) caused by **A)** doxorubicin and **B**) BMS-986094. In the figure legend, the doses indicated with a black outline are the closest to fC_max_. *: p<0.05 and outside the tolerance intervals of vehicle controls. Q: quiescent (cessation of beating).
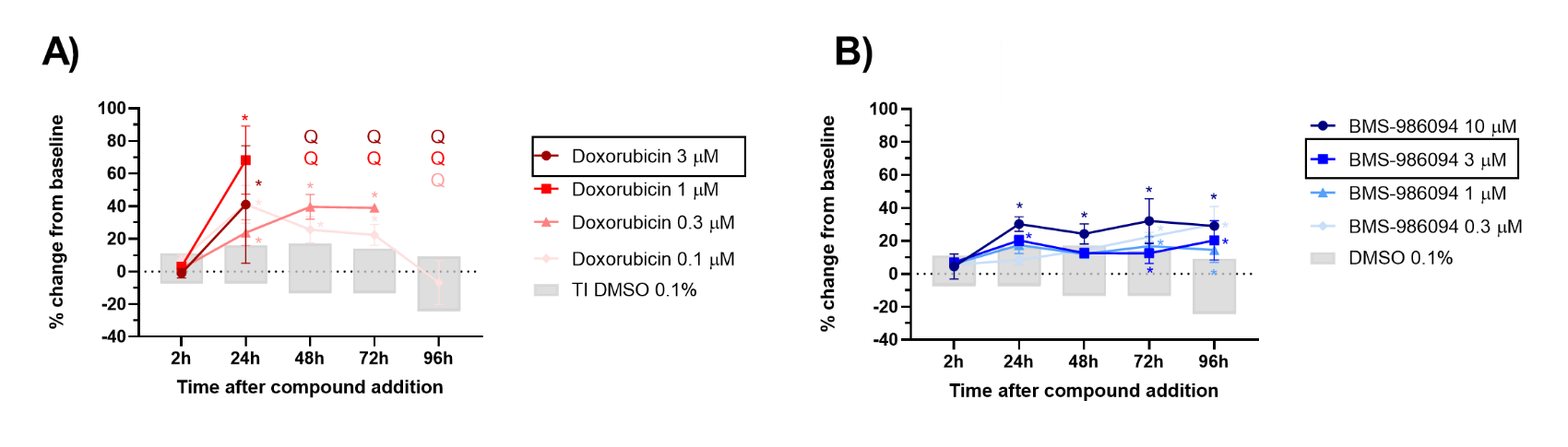


**Supplementary Figure S3:** **Effect of ion-channels trafficking inhibitors on beating rate.** Electrophysiological changes in BR (as mean±SD) caused by **A)** pentamidine, **B**) probucol and **C)** arsenic trioxide. In the figure legend, the doses indicated with a black outline are the closest to fC_max_. *: p<0.05 and outside the tolerance intervals of vehicle controls. Q: quiescence (cessation of beating).


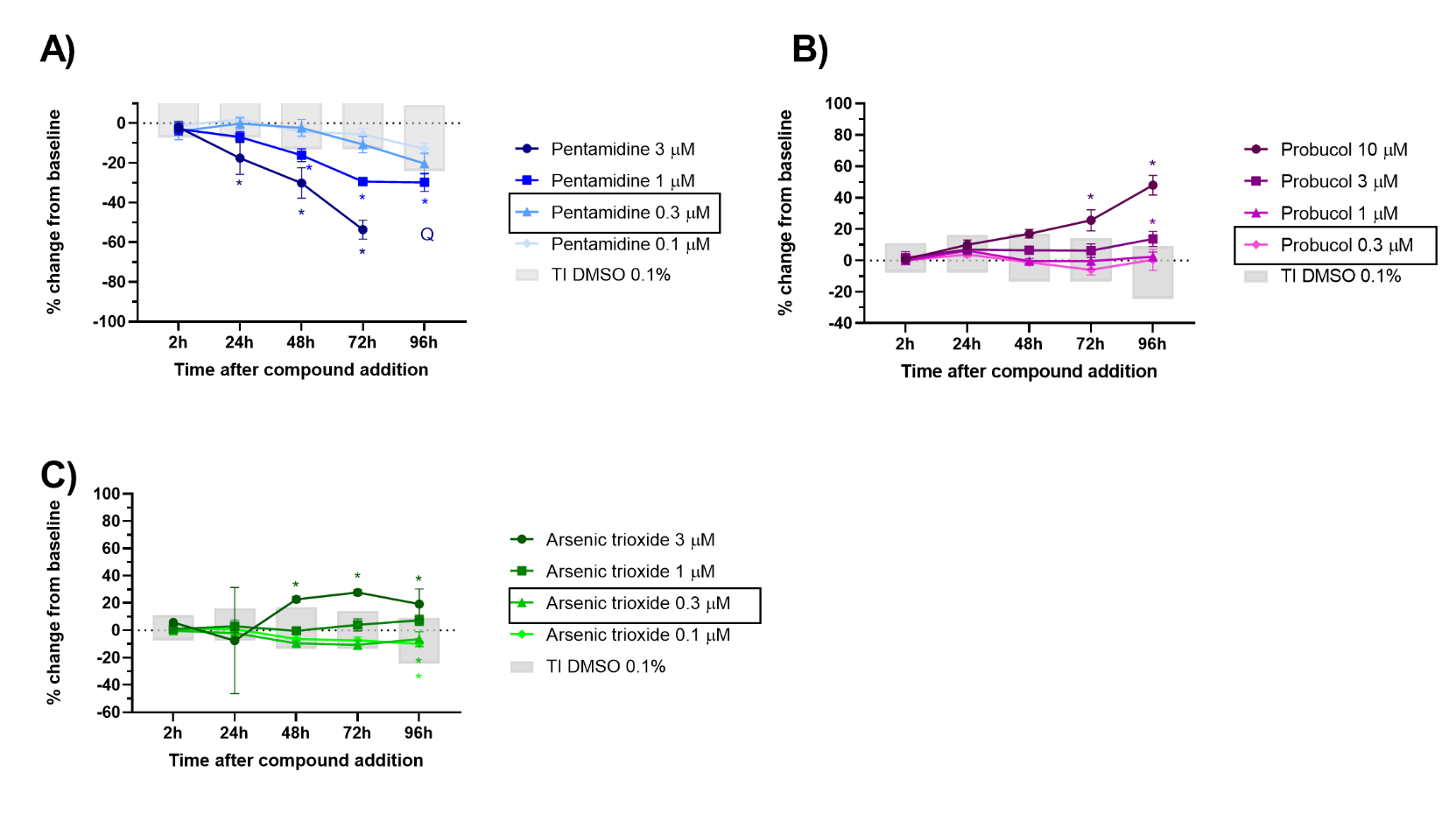


**Supplementary Figure S4:** **Effect of TKis on beating rate.** Electrophysiological changes in BR (as mean±SD) caused by **A)** vandetanib, **B**) nilotinib, **C)** sunitinib and **D)** erlotinib. In the figure legend, the doses indicated with a black outline are the closest to fC_max_. *: p<0.05 and outside the tolerance intervals of vehicle controls. Q: quiescence (cessation of beating).


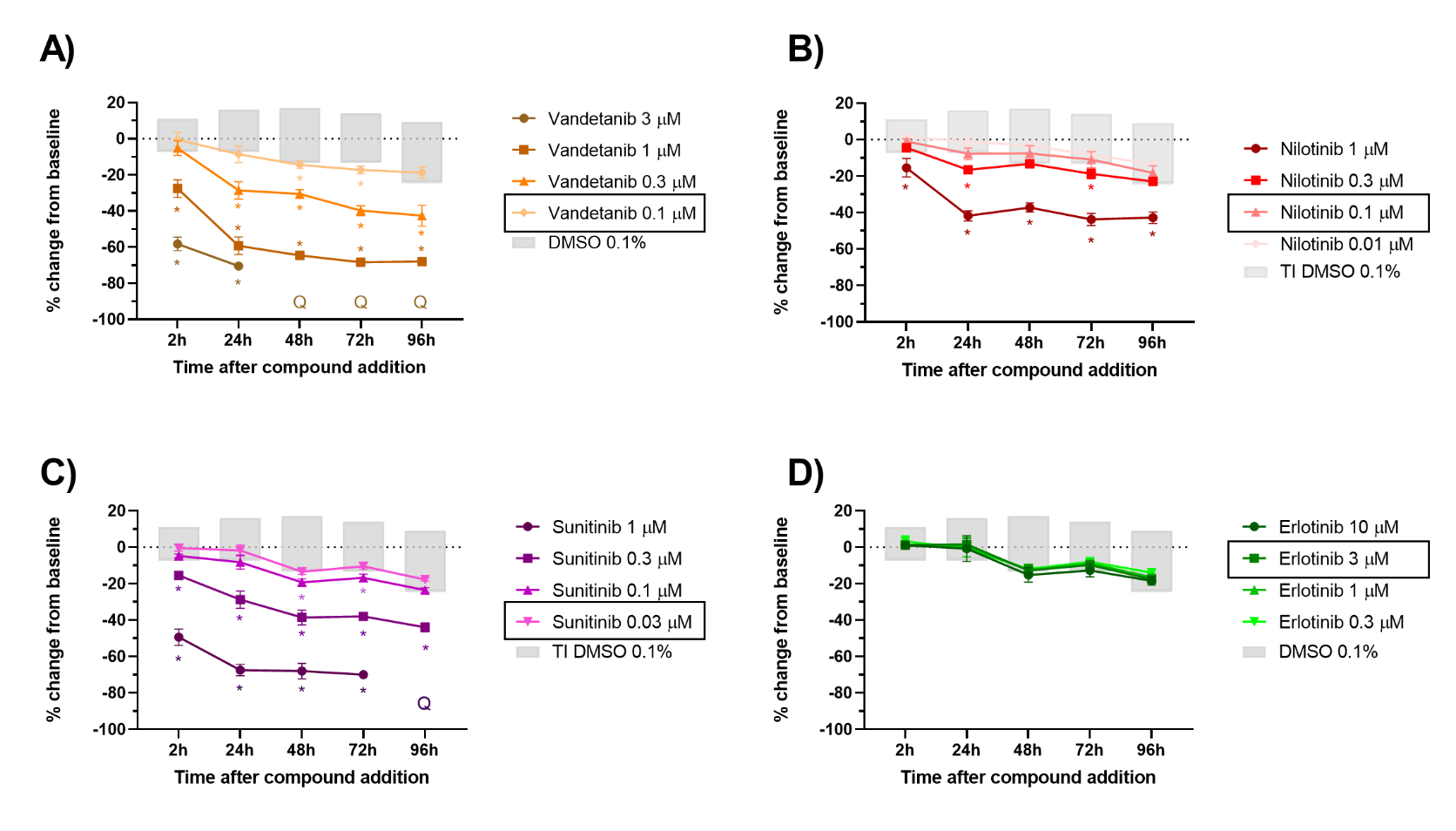


**Supplementary Figure S5:** **Comparison of the sensitivity of functional and cytotoxicity parameters.** Plots depicting at which concentration (as fold over median fCmax, on the x-axis) significant changes the parameters from the functional (FPD, FPDc, BR and Imp) and from cytotoxicity (live cells, annexin, caspases and necrosis) occurred, at 48h time point, for sunitinib and doxorubicin. The grey area represents the range of minimum to maximum fCmax values retrieved from FDA approval packages (see Table 1 and the Methods section in the main text). The dotted line represents the value of median fCmax.
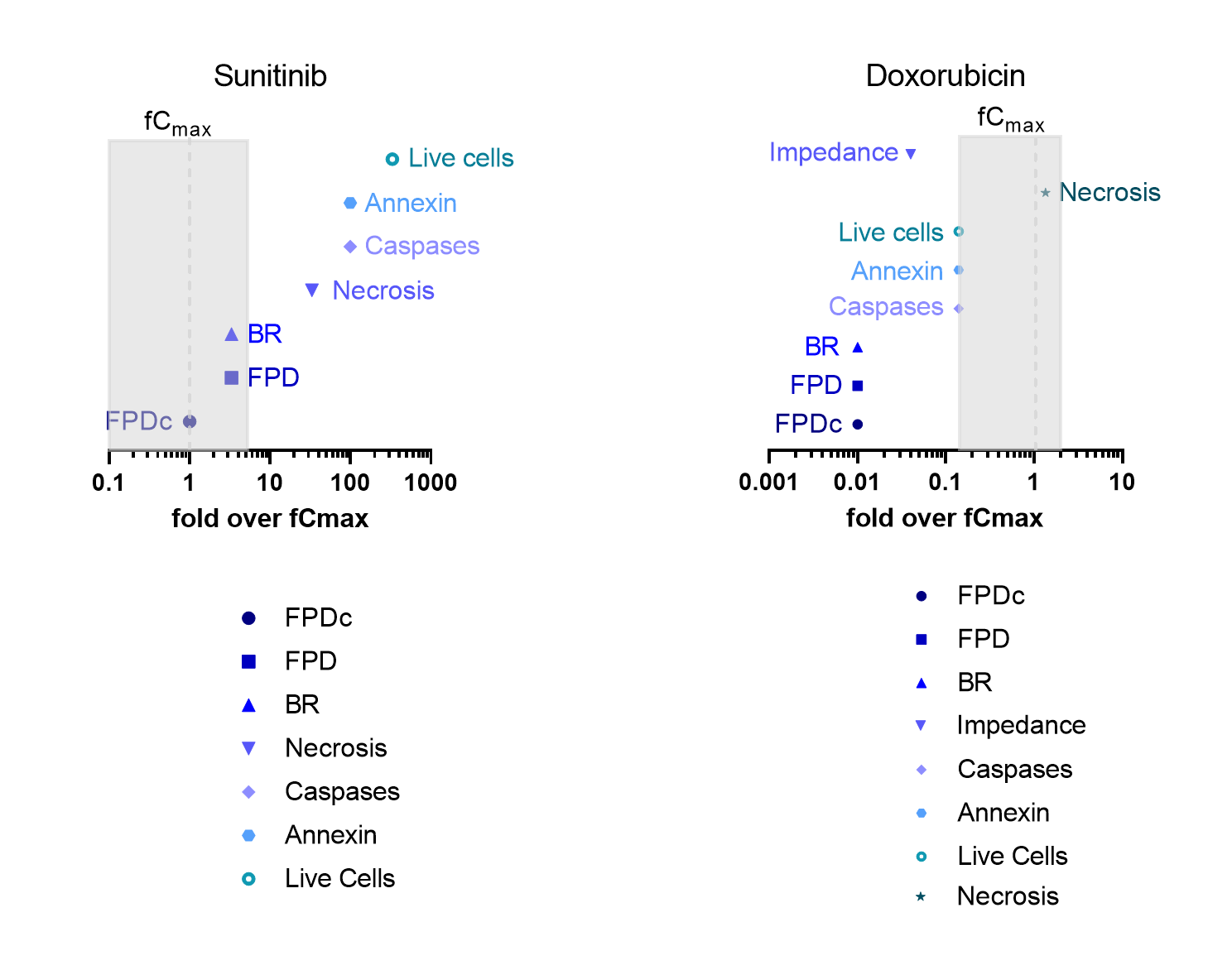

Supplement: Supplementary file 1 [file DataSheet1.docx]
